# Tryptophan Metabolic Enzyme IL4I1 Inhibits Ferroptosis by Decreasing Ubiquitination of Nrf2 via I3P in Glioblastoma

**DOI:** 10.1111/cpr.13816

**Published:** 2025-03-12

**Authors:** Yang Xu, Yu Hong, Tengfeng Yan, Qian Sun, Fanen Yuan, Shanwen Liang, Liguo Ye, Rongxin Geng, Yangzhi Qi, Qingsong Ye, Qianxue Chen

**Affiliations:** ^1^ Department of Neurosurgery Renmin Hospital of Wuhan University Wuhan Hubei China; ^2^ Central Laboratory Renmin Hospital of Wuhan University Wuhan Hubei China; ^3^ Department of Neurosurgery The Second Affiliated Hospital of Nanchang University Nanchang China; ^4^ Centre of Regenerative Medicine Renmin Hospital of Wuhan University, Wuhan University Wuhan China; ^5^ Sydney School of Dentistry The University of Sydney Sydney NSW Australia

**Keywords:** ferroptosis, GBM, IL4I1, indole‐3‐pyruvic acid (I3P), Nrf2, ubiquitination

## Abstract

Glioblastoma multiforme (GBM) is the deadliest brain tumour with an extremely poor prognosis. Tryptophan catabolism could enhance an array of protumour‐genic signals and promoted tumour progression in GBM. However, the mechanisms of oncogenic signalling under tryptophan catabolism and potential therapy targeting this pathway have not been completely understood. Interleukin 4‐induced 1 (IL4I1) is newly defined as a tryptophan metabolic enzyme and the potential function in GBM cells still remains unclear. In our study, we found IL4I1 was upregulated in GBM patients and predicted poor prognosis. Upregulation of IL4I1 inhibited GBM ferroptosis in vitro and in vivo. Further, we found that indole‐3‐pyruvic acid (I3P) from tryptophan mediated by IL4I1 could scavenge free radical and had an impressive role in inhibiting ferroptosis. To clarify the potential mechanism of I3P in GBM ferroptosis, we performed transcriptomic analyses of GBM cells treated with I3P and found that Nrf2 related genes was upregulated. Further, we found that the ubiquitination of Nrf2 could be attenuate by I3P binding with Nrf2 directly. Knockdown of Nrf2 attenuated the induction of anti‐ferroptosis by IL4I1, pointing to Nrf2 as a key mediator of this process. In vivo, overexpression of IL4I1 with ML385 in GBM xenografts promoted ferroptosis. Collectively, this study emphasises the crucial roles of IL4I1 in anti‐ferroptosis through Nrf2 signalling pathway but not AHR pathway by catabolism tryptophan, suggesting IL4I1 and tryptophan reprogramming as potential therapeutic targets for GBM.

## Graphical abstract

Interleukin 4‐induced 1 (IL4I1) is a novel anti‐ferroptosis regulator‐IL4I1 by regulating Nrf2 and serving as a ROS scavenger in glioblastoma multiforme (GBM). In addition, the ubiquitination of Nrf2 could be attenuate by indole‐3‐pyruvic acid binding with Nrf2 directly. Furthermore, IL4I1 is a potential prognostic biomarker and provided a novel anti‐tumour therapeutic target for GBM.

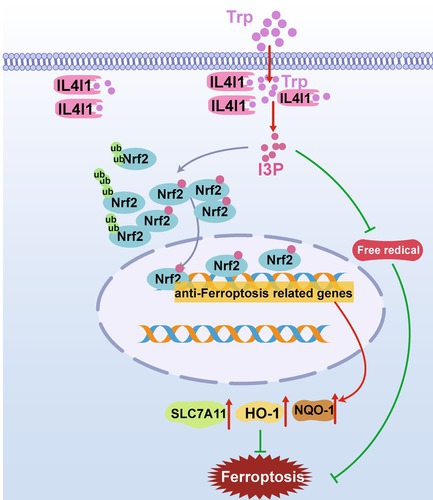

## 1 Introduction

Glioblastoma multiforme (GBM) is the most common and lethal primary malignant tumour in the adult central nervous system. Despite standard treatment with maximal surgical resection and adjuvant radio‐chemotherapy, the 1‐ and 5‐year survival rates of patients are only 42.5% and 6.8%, respectively [1]. Dysregulation of programmed cell death is an important factor for poor diagnosis and therapeutic resistance [2]. Ferroptosis is the most enriched programmed cell death process with consistent prognostic value in GBM [3]. In addition, ferroptosis is a form of regulated cell death that mainly relies on iron‐mediated oxidative damage and subsequent cell membrane damage [4]. Moreover, GBM is more suitable for pro‐ferroptotic therapy because of its highly expression of ferroptosis‐related genes [5]. However, the sulfasalazine that inhibits SCL7A11, has been investigated clinically as monotherapy in patients with glioma and in combination with radiosurgery in patients with GBM, but has only demonstrated marginal efficacy [6, 7]. Ferroptosis can be regulated at the epigenetic, transcriptional, and posttranscriptional levels [8]. Thus, it is urgent to identify more specific ferroptosis‐associated targets, which may lead to the development of GBM treatments.

Metabolism reprogramming, which is involved in ferroptosis, is one of the hallmarks of cancer [9]. Recently, amino acid reprogramming demonstrated important roles in biological processes in GBM, such as energy regulation and immunomodulation [10, 11, 12]. Numerous recent studies have successfully constructed gene risk signatures based on the amino acid metabolism of gliomas, which could predict clinical features including overall survival and immune cell infiltration in the tumour microenvironment (TME) [13, 14]. Tryptophan is an essential amino acid for humans. The levels of tryptophan metabolites were significantly higher in primary GBM cell lines than those in human astrocytes [15]. Recently, Panitz et al. revealed that the serum levels of tryptophan and its metabolites are significantly lower in patients with GBM than those in healthy controls. Furthermore, tryptophan metabolism‐related genes were expressed in almost all GBM subtypes according to single‐cell sequencing analysis [16]. These findings indicated the importance of tryptophan metabolism in GBM. Considering the latest reports showing that tryptophan metabolites are also involved in ferroptosis regulation, an in‐depth investigation of these findings will hopefully shed new light on the pro‐ferroptotic therapy of GBM [17].

Tryptophan metabolism is mainly mediated by tryptophan‐catabolic enzymes (TCEs) in tumour cells. The metabolites promote GBM progression by regulating tumour cells and the TME [18]. Interleukin 4‐induced 1 (IL4I1) is a newly defined TCE, known as L‐amino acid oxidase, mainly implicated in immune regulatory functions [19]. Recently, it has been demonstrated that IL4I1 could catabolise tryptophan into indole‐3‐pyruvic acid (I3P), which could enhance AHR nuclear translation and transcription. Further, among the I3P‐derived metabolites, kynurenic acid (KynA) and indole‐3‐aldehyde (I3A) lead to the activation of the AHR signalling pathway, which might increase the migratory and invasive abilities of tumour cells and reduce CD8+ T cell proliferation [20]. Interestingly, the IL4I1/I3P axis was proposed to regulate ferroptosis both through a radical scavenging mechanism and by orchestrating a gene expression profile that attenuates ferroptosis [21]. However, the specific regulatory mechanism of the IL4I1/I3P axis in GBM ferroptosis was unclear. Therefore, it is urgent to elucidate the regulatory mechanism underlying the effects of IL4I1 on GBM ferroptosis and evaluate its clinical value as a novel therapeutic target.

In the current study, we identified IL4I1 as an oncogene in GBM that could predict patient prognosis. Furthermore, we found that IL4I1‐mediated tryptophan metabolism inhibited GBM cell ferroptosis in vitro and in vivo. Specifically, among the metabolites generated from tryptophan via IL4I1, only I3P played a critical anti‐apoptotic role via an AHR‐independent way, including direct activation of the Nrf2 pathway and free radical scavenging. These findings revealed that tryptophan catabolism not only regulates the immune microenvironment but also participates in the regulation of ferroptosis. Collectively, we identified IL4I1 as a novel anti‐ferroptotic regulator in GBM, which could be used to develop an innovative therapeutic strategy.

## 2 Materials and Methods

### 2.1 Bioinformatic Analysis of The Cancer Genome Atlas Database

The expression profiles of IDO1, TDO2, and IL4I1 in different glioma grades and pathological GBM subtypes as well as the survival analysis of these three genes in mesenchymal subtype GBM were obtained from the GlioVis portal website (https://gliovis.bioinfo.cnio.es) [22]. We also downloaded GBM data from The Cancer Genome Atlas (TCGA) database and used R to analyse the relationship between overall survival and these three genes in all patients with GBM [14].

### 2.2 Clinical Glioma Samples and Control Brain Tissues

Glioma samples were collected during the surgery. Some clinical samples were stored at −80°C and others were used to prepare paraffin sections. Control brain tissues were acquired from patients with traumatic brain injury during the emergency surgery. Glioma diagnosis was provided by the pathologists of the Department of Pathology at the Renmin Hospital of Wuhan University according to the 2021 WHO classification [23]. The IDH status of GBM samples in this study was wild type (WT). Patient clinical information is listed and presented in Supplemental Tables S1 and S2. The study was approved by the Renmin Hospital of Wuhan University's Institutional Ethics Committee of the Faculty of Medicine (approval number: WDRY2021‐K109) and informed consent was obtained from all participants.

### 2.3 RNA Extraction and Quantitative Real‐Time PCR

Trizol reagent (Servicebio, Wuhan, China) was used to extract the total RNA of clinical samples. The PrimeScript RT Reagent Kit (Servicebio, Wuhan, China) was used to synthesise cDNA. SYBR quantitative PCR (qPCR) SuperMix plus (NovoStart, Jiangsu, China) was used to detect the relative expression levels of IL4I1 following the manufacturer's instructions. Detection was performed using the 2.1 real‐time PCR Systems of Bio‐Rad CFX Manager (Bio‐Rad, USA). The comparative Ct method was used to analyse the relative gene expression levels in clinical samples, and actin was used as internal control. Primer sequences are listed in Supplemental Table S3.

### 2.4 Cell Lines and Media

The 293 T, U87, and U251 cells lines, purchased from the Cell Bank of the Shanghai Institute of Biochemistry and Cell Biology (Shanghai, China), were authenticated by STR profiling and free of mycoplasma contamination. These cell lines were described in our previous study [24]. Cells were cultivated in high glucose DMEM, and 10% foetal bovine serum supplemented with 1% penicillin/streptomycin. The cell culture conditions were 37°C and 5% CO_2_. Furthermore, a special medium with DMEM without added tryptophan was prepared.

### 2.5 Cell Transfection

A lentivirus, which was constructed by Genechem (Shanghai, China) using GV260, was used to overexpress IL4I1 in U87 and U251 cells. The lentivirus was added in the medium following the manufacturer's instructions to infect GBM cells for 24 h. Subsequently, 2 μg/mL puromycin was used to select stably infected cells. The stable pooled clones were verified using western blot.

### 2.6 shRNA Knockdown

U87 and U251 cells were transfected with AHR or Nrf2 shRNA using liposomal Transfection Reagent (Yeasen, Shanghai, China) following the manufacturer's instructions. shRNAs specifically targeting AHR and Nrf2 were purchased from Tsingke Biotechnology (Beijing, China).

### 2.7 Cell Death Analysis

Cells were seeded in 96‐well plates and ferroptosis inducers (10 mM erastin or 1 mM RSL3) were added into the medium 24 h after plating. Cell death was monitored using the CellTox Green Cytotoxicity Assay, which preferentially stains the DNA of dead cells. When the dye binds DNA in compromised cells, the dye's fluorescent properties are substantially enhanced. The images were captured using an Olympus IX71 microscope (Olympus, Japan).

### 2.8 Lipid Peroxidation Analysis

BODIPY 581/591 C11 (Invitrogen, USA) is a lipid peroxidation sensor, which was used to assess ROS levels and lipid peroxidation in cells after treatment with CytoFlex (Beckman, US) following the manufacturer's instructions. All data were analysed using Flowjo 10.4.

### 2.9 ROS Detection Assay

To assess the ROS levels, we used the fluorescent probe DCFH‐DA (Beyotime, Shanghai, China) according to the manufacturer's instructions. Intracellular ROS oxidised non‐fluorescent DCFH to generate fluorescent DCF. Detection of DCF fluorescence could be used to measure the level of intracellular ROS. The fluorescence of cells after treatment was detected using CytoFlex (Beckman, US) and all data were analysed using Flowjo 10.4.

### 2.10 Glutathione/Oxidised Glutathione Assay

The intracellular levels of glutathione (GSH) were measured using a GSH/oxidised glutathione (GSSG) Assay Kit (Beyotime, Shanghai, China). According to the manufacturer's instructions, the treated cells after treatment were collected and protein concentrations were quantified using the BCA method. The total GSH levels were measured using the enzymatic recycling method using glutathione reductase and 5′,5′‐dithio‐bis (2‐nitrobenzoic acid). The sulfhydryl group of GSH reacts with DTNB and produces a yellow‐coloured 5‐thio‐2‐nitrobenzoic acid, which has an absorbance in the range of 405–414 nm. GSSG levels were accomplished by first derivatising GSH with 2‐vinylpyridine. The concentrations of reduced GSH were calculated by subtracting the GSSG levels from those of the total GSH (GSH = total GSH − 2 × GSSG). The intracellular GSH levels were determined based on the cellular protein concentrations.

### 2.11 DPPH Scavenging Assay

Radical scavenging activity of tryptophan‐derived metabolites was accessed using 1,1‐diphenyl‐2‐picrylhydrazyl (DPPH) (D9132, Sigma)—the stable free radical. Ferrostatin‐1 which could inhibit ferroptosis was used as the positive control group [25]. All compounds (200 μM) were added to a 200 mM DPPH solution in pure methanol. After 10 min incubation, the absorbance was measured at 517 nm. The decrease in absorbance due to radical scavenging was calculated relative to that of the H_2_O control.

### 2.12 Immunofluorescence Assays

Treated cells were fixed with 4% paraformaldehyde and permeabilised using 0.1% Triton‐X100. After washing in PBS thrice, cells were blocked with 1% BSA for 30 min. Then, they were incubated with an anti‐Nrf2 antibody overnight at 4°C followed by incubation with fluorescent secondary antibodies (ABclonal, China) for 1 h at room temperature (RT). The samples were coverslipped with DAPI‐containing antifade mountant (ANT046, Antgene, China). Images were captured using an Olympus BX53 microscope (Olympus, Japan) and a 40× objective.

### 2.13 Immunohistochemistry Staining

Paraffin sections were prepared from the collected tissues and gradient hydration, heat‐mediated antigen retrieval, and endogenous peroxidase removal were performed. Sections were blocked with 1% BSA for 1 h, followed by incubation with primary antibodies overnight at 4°C. Then, tissues were incubated with HRP‐labelled secondary antibodies (ABclonal, China). DAB (Servicebio, China) was applied for dyeing while haematoxylin was applied for nuclear staining. Images were captured using an Olympus BX53 microscope (Olympus, Japan). The distribution and intensity of IL4I1 staining were assessed using a semi‐quantitative method (0 = negative, 1 = weak, 2 = moderate, 3 = strong, 4 = strong and widely distributed).

### 2.14 Construction of the Xenograft Model and Tumour Measurement

Nude mice were randomly allocated to experimental groups using a random number table. U87 cells stably expressing luciferase or luciferase‐IL4I1 (1.0 × 10^5^ cells/μL) were injected into the frontal lobes of male nude mice (4–5‐week‐old) and implantation was achieved using a stereotactic instrument (*n* = 10/group). The tumour volume was assessed using bioluminescence imaging at days 7 and 21 (IVIS Lumina III; PerkinElmer). When serious neurological symptoms appeared and/or an evident weight loss occurred, the survival time was recorded for survival analysis and the brain with the tumour was removed. Further, U87 cells with luciferase or luciferase‐IL4I1 (4 × 10 [6]) were injected subcutaneously into the axilla of 5‐week‐old nude mice (*n* = 5/group). Then, 10 days after cell planting, the mice were treated with ML385 (30 mg/kg) and erastin (30 mg/kg) by intraperitoneal injection every 3 days. After three drug treatments, the tumour size was monitored using bioluminescence imaging (IVIS Lumina III; PerkinElmer) at days 19. Mice were euthanised at 28 days after cell injection to obtain tumour weights. All samples were fixed in 4% paraformaldehyde for further analysis. No blind method is involved for the animal experiments. All procedures and experiments on animals were approved by the Institutional Animal Care and Use Committee subordinate to Renmin Hospital of Wuhan University (WDRM 20201111).

### 2.15 RNA‐Seq Analysis

U87 cells were treated in triplicates with 100 mM I3P in DMEM medium for 24 h. DMEM medium was used as the control treatment. After the treatment, total RNA was extracted using Trizol. For RNA‐seq, library construction and sequencing were performed by HaploX Biotechnology (Jiangxi, China) following the standardised flow. The libraries were sequenced on an Illumina Nova‐seq 6000 platform according to the manufacturer's instructions. The Volcano Plot and heat map of differentially expressed genes (DEGs) were constructed using R. Genes with an adjusted *p* value of < 0.05 were considered to be differentially expressed.

### 2.16 Western Blotting

RIPA buffer containing cooktiles (protease inhibitors; Beyotime) and PMSF (phosphatase inhibitors; Beyotime) was used to lyse cells or tissues for 30 min at 4°C. Nuclear and cytoplasmic proteins were extracted using the Nuclear and Cytoplasmic Protein Extraction Kit (Beyotime, China) [26]. After using the BCA kit (Biosharp, China) to determine the protein concentration, proteins were separated using SDS‐PAGE and then transferred onto PVDF membranes. Membranes were blocked with 5% skim milk for 1 h at RT, and incubated with primary antibodies overnight at 4°C, followed by incubations with the corresponding secondary antibodies for 1 h at RT. A Bio‐Rad system was utilised to scan the blots and detect the grey value of the blots. The relative protein quantity was assessed using ImageJ and normalised to that of actin.

### 2.17 Schrödinger Ligand Docking

The structures of ligands were downloaded from the PubChem website (https://pubchem.ncbi.nlm.nih.gov), and then the structures were optimised by the LigPrep tool. The sequence of proteins was retrieved from the RCSB database (https://www.rcsb.org/). Molecular docking of the proteins and ligands were carried out by using Maestro 11.5 version. First, the protein was optimised before docking using the Protein Preparation Wizard, and the ligand was prepared with LigPrep tool. Further, partial atomic charges attribution, protonation states generation at pH 7 ± 2.0 and energy minimisation were achieved using OPLS‐2005 force field. To test the docking parameters, all ligands were docked into the catalytic pocket of the protein using Grid‐Based Ligand Docking with Energetics (Glide v11.5, Schrödinger) in “extra precision” mode without applying any constraints. PyMol2.4 was used to visualise the composite PDB format file.

### 2.18 MST Binding

Human NRF2, His‐tagged recombinant human NRF2 was labelled with RED‐tris‐NTA 2nd generation dye following manufacturers protocol. Then, 150 μL of 200 nM Human NRF2 in PBS‐T (PBS, pH 7.4, 0.005% Tween‐20) was incubated with 150 μL of 100 nM dye in PBS‐T for 30 min at RT. After a centrifugation for 10 min at 15,000*g*, 4°C labelled protein was diluted in PBS‐T to a final concentration of 100 nM in labelled was labelled with red labelled was to a final concentration of 100 nM. The interactions between the antibodies and their binding partners were measured in Monolith NT.115 Standard Treated Capillaries. The measurements were performed in buffer with 1%DMSO PBS pH 7.4. Before the MST measurements, samples were centrifuged. The ligands for the binding studies were dissolved in target at double the concentration. The measurements were performed on a NanoTemper Technologies Monolith NT.115 instrument. The samples were measured at Medium MST power and LED power of 100%. The data were analysed using MO. Affinity Analysis Software.

### 2.19 Reagents and Antibodies

The compounds used in our study included: L‐tryptophan (HY‐N0623, MCE, USA); I3P (I7017, Sigma, USA); indole‐3‐acetic acid (IAA; HY‐18569, MCE, USA); indole‐3‐latic acid (ILA; HY‐113099, MCE, USA); I3A (HY‐W007376, MCE, USA); KynA (HY‐100806, MCE, USA); erastin (HY‐15763, MCE, USA); RSL3 (HY‐100218A, MCE, USA); and Ferrostatin‐1 (HY‐100579, MCE, USA). The antibodies used in our study included: actin (AC026, ABclonal, China); IL4I1 (PH0426, Abmart, China); Nrf2 (380773, Zenbio, China); SLC7A11 (R26116, Zenbio, China); HO‐1 (R24541, Zenbio, China); NQO‐1 (11451‐1‐AP, Proteintech, China); AHR (A1451, ABclonal, China); Histone H3 (17168‐1‐AP, Proteintech, China); HRP Goat Anti‐Rabbit IgG (AS014, ABclonal, China); HRP Goat Anti‐Mouse IgG (AS003, ABclonal, China); and Cy3 Goat Anti‐Rabbit IgG (AS007, ABclonal, China).

### 2.20 Statistical Analysis

SPSS 23.0 and GraphPad Prism 8 were employed for statistical analysis. Student's t test was performed for data analysis between two groups while one‐way analysis of variance followed by Student–Newman–Keuls post hoc test was performed when there were more than two groups. All data are expressed as means ± standard deviations (SD). A *p* value of < 0.05 was considered statistically significant.

## 3 Results

### 3.1 IL4I1, an Important TCE, Was Upregulated and Related to Poor Outcomes in GBM

**FIGURE 1:**
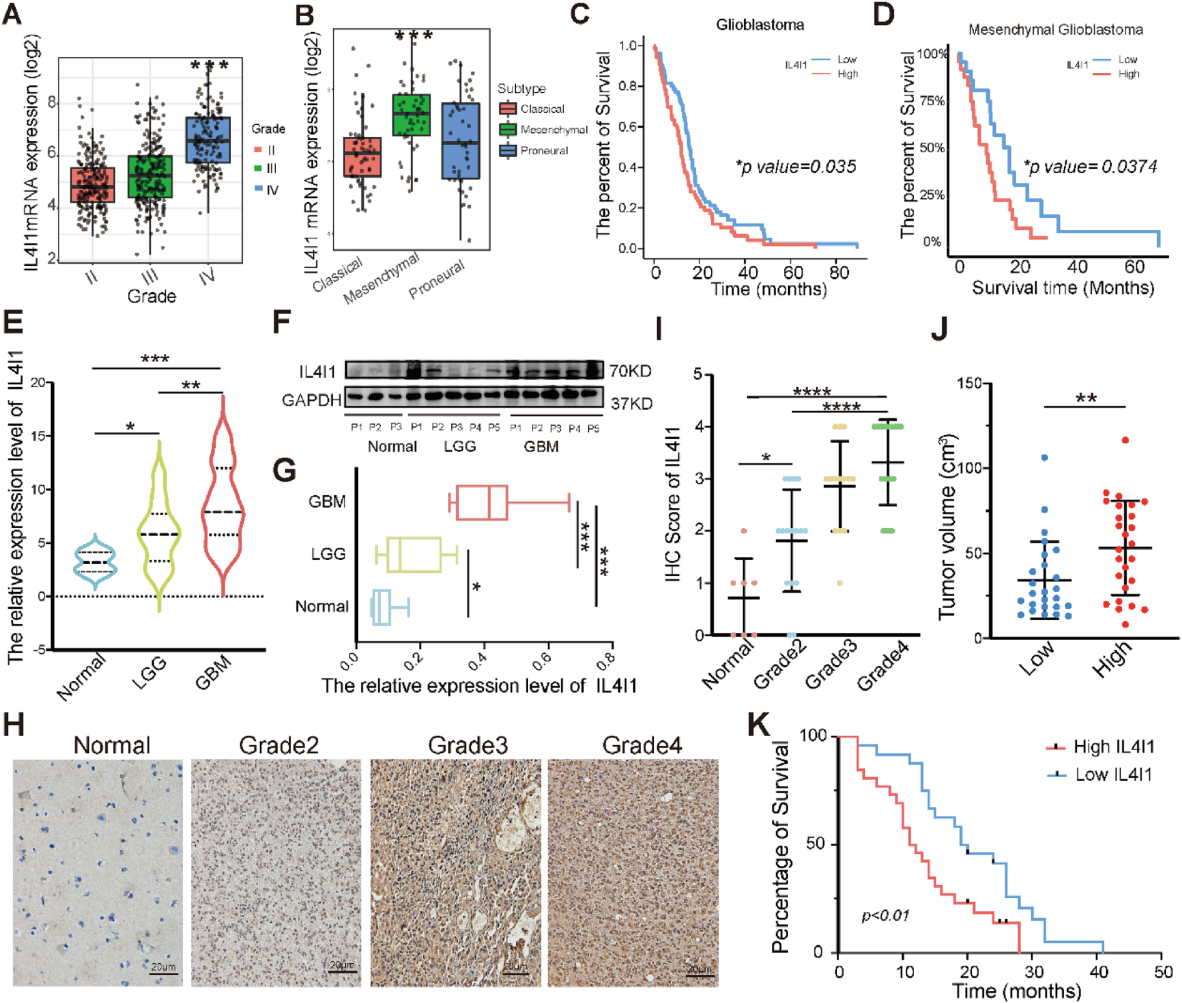
IL4I1 was upregulated and related to poor outcomes in GBM patients. (A) Expression of IL4I1 in human gliomas according to the TCGA database. (B) Comparison of IL4I1 expression levels between GBM MES, PN, or CL subtypes. Boxplots indicate the median quartiles, with whiskers extending the minimum and maximum range. (C) Kaplan–Meier curves of the overall survival of GBM patients in TCGA divided into groups of high and low IL4I1 expression. (D) Kaplan–Meier curves of the overall survival of MES‐type GBM patients in TCGA divided into groups of high and low IL4I1 expression. (E) Real‐time qPCR for IL4I1 mRNA in GBM (*n* = 51), LGG (*n* = 22), and non‐tumour tissues (*n* = 6). (F, G) Western blot for IL4I1 protein in GBM (*n* = 10); LGG (*n* = 10); and non‐tumour tissues (*n* = 6). (H) Representative IHC staining images for IL4I1 in clinical tissues. Grades II–IV indicate the pathologic grades of the glioma samples. Scale bars, 20 μm. (I) IHC score of IL4I1 in clinical tissues. The IHC scores were graded as 0, 1, 2, 3, and 4. Non‐tumour tissue, *n* = 7; WHO II, *n* = 16; WHO III, *n* = 14; and WHO IV, *n* = 19. (J) Tumour volume of GBM patients with high or low level of IL4I1 expression in tumours. (K) Kaplan–Meier analysis for GBM patients with high or low level of IL4I1 expression in tumours. *p < 0.05, **p < 0.01, ***p < 0.001, ****p < 0.0001.

Most of the free tryptophan in tumour cells is catabolised by TCEs and plays an important role in the progression of various cancers [18]. The TCE expression level is also correlated with the efficacy of immunotherapy in GBM patients [27]. To further investigate the correlation between TCE expression and patient clinical features, we screened out three classical TCEs, IDO1, TDO2, and IL4I1, and performed bioinformatic analysis using TCGA database. The mRNA expression levels of these three genes were positively correlated with glioma malignancy (Figure 1A, Figure S1A,E). Meanwhile, higher expression of TCEs was also observed in IDH WT gliomas compared with IDH mutant gliomas (Figure S1I). Furthermore, we detected the mRNA expression of TCEs in the three major GBM subtypes and found that their levels were significantly higher in the mesenchymal subtype, compared with those in the classical and pro‐neural subtypes (Figure 1B, Figure S1B,F). Resistance to chemotherapy and highly malignant invasiveness are the representative features of mesenchymal GBM [28]. However, among the three TCEs, only the mRNA level of *IL4I1* was closely related to prognosis in patients with gliomas (Figure 1C, Figure S1C,G) and mesenchymal type GBM (Figure 1D, Figure S1D,H). To verify these results, we performed a series experiment in our clinical samples. We performed PCR in clinical samples including six normal brain tissues, 22 low grade gliomas (LGG), and 51 GBMs and found that the expression level of IL4I1 in GBM was significantly higher compared with that in normal brain tissues and LGG (Figure 1E). This was also confirmed on the protein level using western blot and IHC assays (Figure 1F–I). Furthermore, the tumour volume in MRI was significantly larger in the group with higher *IL4I1* levels compared with that in the other groups (Figure 1J). Finally, Kaplan–Meier analysis showed that higher *IL4I1* levels could predict the poor prognosis of GBM patients (Table 1, Figure 1K). Thus, IL4I1 is closely related to GBM malignancy and may serve as a survival predictor and therapeutic target. Hence, we further explored the potential function of IL4I1 in GBM.

**TABLE 1:** Kaplan–Meier analysis of 51 clinical GBM patients.

Clinicopathological variables	Number	Average survival time	*p*
All cases	51	16.830 ± 1.420	
Age at diagnosis (years)			0.960
< 55	21	16.849 ± 2.273	
≥ 55	30	16.814 ± 1.806	
Gender			
Male	24	19.346 ± 2.229	0.151
Female	27	14.611 ± 1.816	
Lobe lesions			0.816
1	39	16.734 ± 1.470	
≥ 2	12	17.125 ± 3.777	
Volume (cm^3^)			0.067
> 40	25	14.520 ± 1.620	
< 40	26	19.039 ± 2.240	
IL4I1			0.002**
High	25	12.690 ± 1.553	
Low	26	20.685 ± 2.052	

*Note:* **p < 0.01.

### 3.2 Overexpression of IL4I1 Protected Against Ferroptosis of GBM Cells In Vitro

**FIGURE 2:**
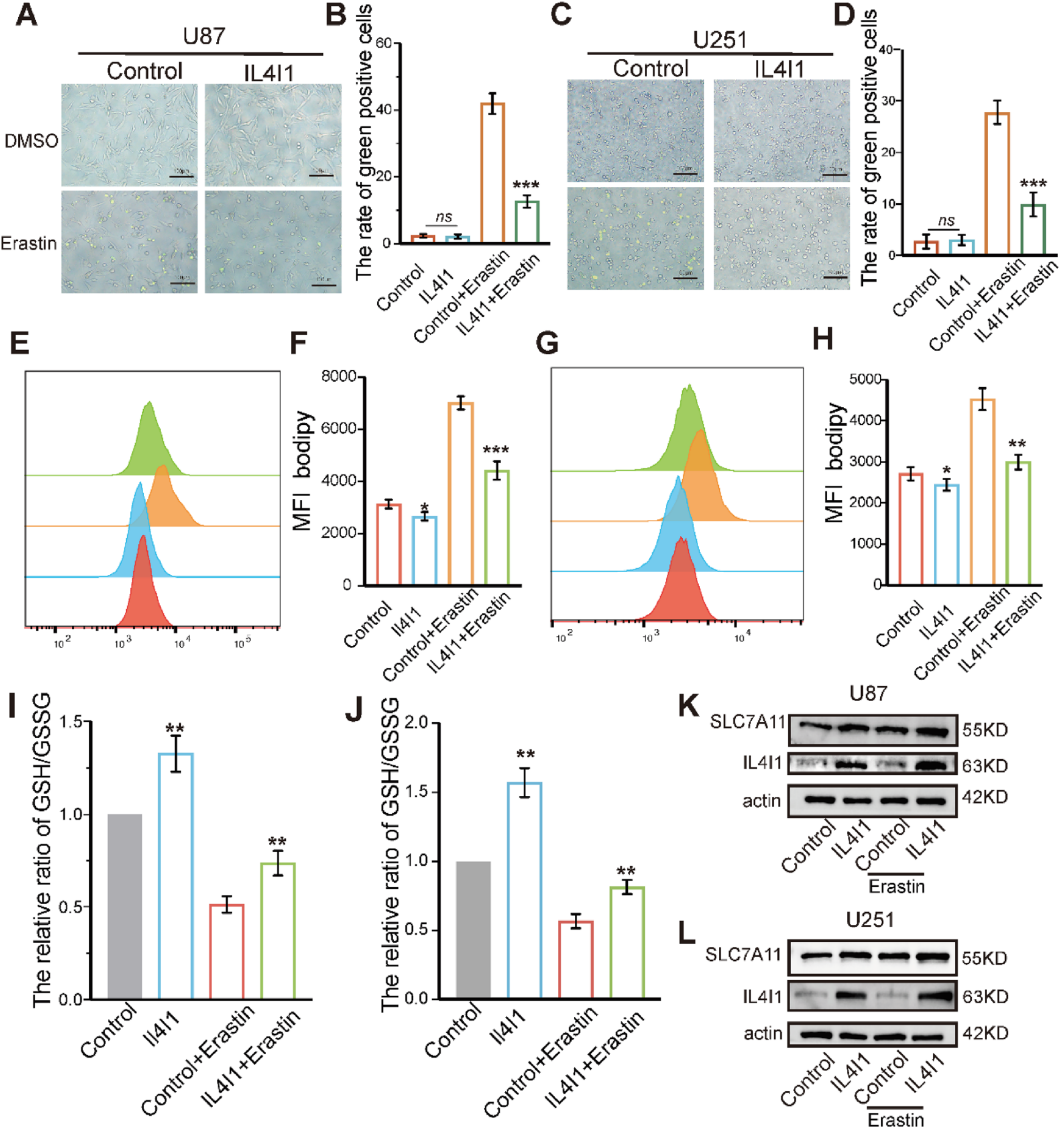
Overexpression of IL4I1 protected against ferroptosis of GBM cells in vitro. (A–D) IL4I1 inhibited erastin‐induced ferroptosis in U87 cell and U251 cells. (E–H) IL4I1 decreased lipid peroxidation induced by erastin determined by flow cytometry using C11‐BODIPY in U87 and U251 cells, all error bars represent standard deviation. (I, J) IL4I1 increased GSH/GSSG ratio in U87 and U251 cells treated with erastin for 24 h relative to untreated control. (K, L) Effects of IL4I1 overexpression on the levels of ferroptosis‐related proteins in GBM cells. All error bars represent standard deviation. *p < 0.05, **p < 0.01, ***p < 0.001, ****p < 0.0001.

Tryptophan plays a crucial role in supporting cell survival and is likely involved in ferroptosis [17, 29]. To investigate the relationship between IL4I1 and ferroptosis, we conducted functional studies to access ferroptosis in U87 and U251 cells which overexpressing IL4I1. First, we monitored the cell death using Cell Tox green after adding DMSO and ferroptosis inducers into the medium respectively. The results showed that IL4I1 prevented U87 and U251 cells from erastin and RSL3‐induced ferroptosis (Figure 2A–D, Figure S2A–D). Second, through measuring with C11‐BODIPY via flow cytometry after erastin and RSL3 treatment, we found that overexpression of IL4I1 significantly decreased lipid peroxidation of U87 and U251 cells (Figure 2E–H, Figure S3A–D). Massive lipid peroxidation is a hallmark of ferroptosis [30]. Reduced GSH, which can be transferred to GSSG, is a central player in ferroptosis [31] Generally, the ratio of GSH/GSSG was used as a reflection of the strength of ferroptosis. IL4I1 overexpression increased the ratio of GSH/GSSG, indicating that upregulated IL4I1 played an important role in ferroptosis inhibition (Figure 2I,J, Figure S4A,B). Finally, erastin targeted the cystine/glutamate antiporter system xc which consisted of SLC7A11 and SLC3A2 [32]. Consistently, the expression level of SLC7A11 was also elevated in the IL4I1 group compared with that in the control group in U87 and U251 cells according to immunoblotting results (Figure 2K,L). Collectively, these results suggested that IL4I1 inhibited ferroptosis in GBM cells.

### 3.3 IL4I1 Overexpression Inhibited Ferroptosis and Reduced Overall Survival in Orthotopic GBM Mouse Xenografts

**FIGURE 3:**
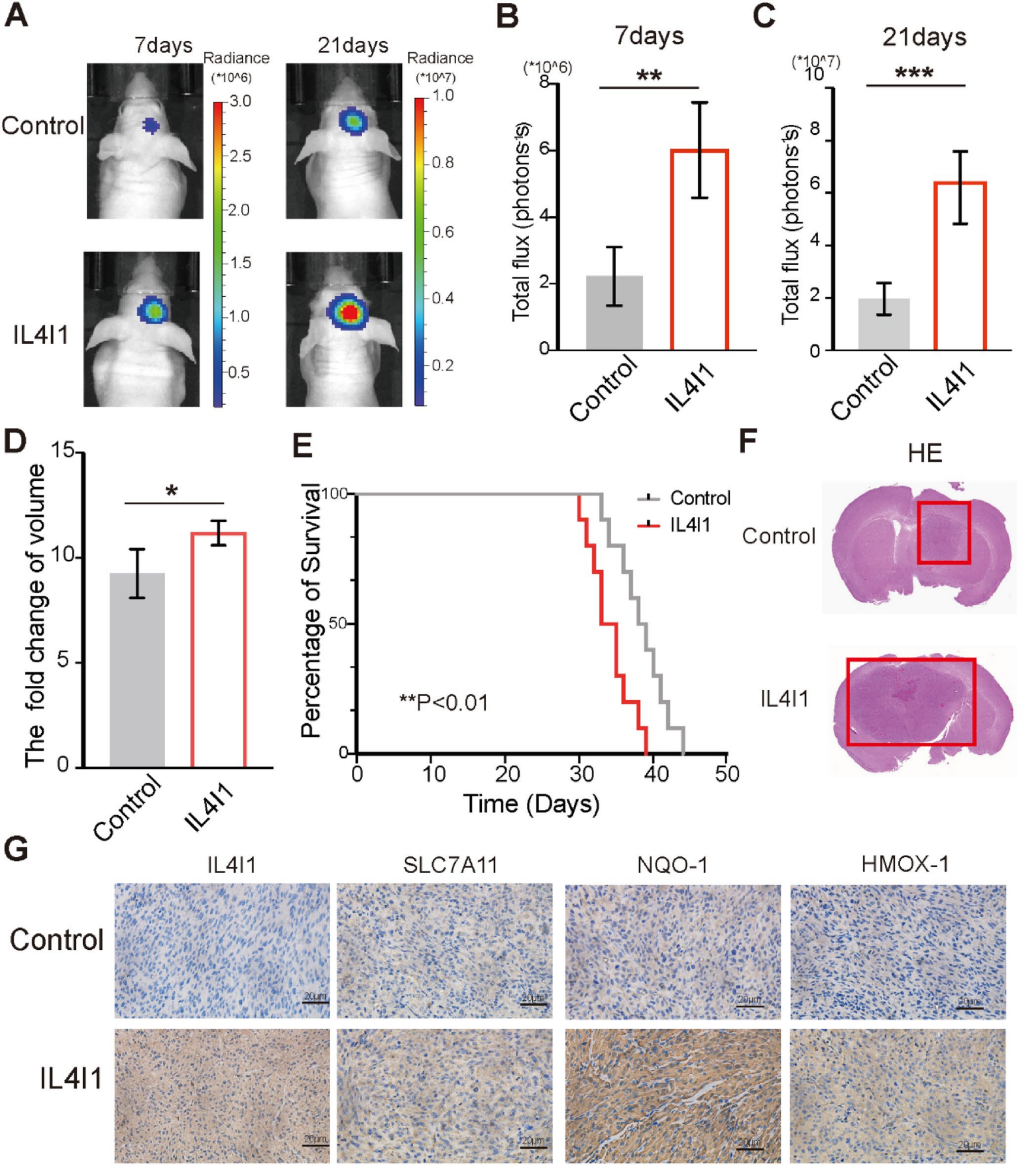
IL4I1 overexpression inhibited ferroptosis and reduced overall survival in orthotopic GBM mouse xenografts. (A) IL4I1 enhanced the growth of GBM in the intracranial xenograft model. Luciferase fluorescence of mouse xenografts was detected by an IVIS at 7th and 21st days after tumour injection. (B–D) Quantification of luciferase fluorescence (*n* = 4). (E) Mouse survival is shown by Kaplan–Meier curves (control group, *n* = 12; IL4I1 group, *n* = 12). *P* values were calculated using the log‐rank test. (F) Representative images of mouse brain tumour HE sections. (G) IHC analyses of IL4I1, SLC7A11, NQO‐1, and HOMX‐1 in mouse tumour sections. Scale bars, 20 μm. All error bars represent standard deviation. *p < 0.05, **p < 0.01, ***p < 0.001.

After demonstrating the function of IL4I1 in vitro, we investigated how IL4I1 influenced the biological behaviour of GBM in vivo. We used stably lentivirus‐transfected U87‐luc and U87‐luc‐IL4I1 cells to construct orthotopic GBM mouse xenografts. Bioluminescence imaging at days 7 and 21, respectively, showed that the tumour grew significantly faster in the IL4I1‐overexpression group compared with the control group (Figure 3A–D). Survival analysis showed that the mouse xenografts in the IL4I1‐overexpression group died remarkably earlier than those of the control group (Figure 3E). HE staining also revealed that larger and more blood vessels infiltrated the tumour in the IL4I1‐overexpression group (Figure 3F), suggesting more malignant biological processes in the IL4I1‐overexpression group. Next, IHC results showed that ferroptosis‐related genes, including SLC7A11, NQO‐1, and HMOX‐1, were significantly upregulated when IL4I1 was overexpressed (Figure 3G). These findings demonstrated that IL4I1 promoted tumour proliferation and enhanced ferroptosis resistance.

### 3.4 IL4I1‐Driven Tryptophan Metabolites Suppress Ferroptosis of GBM Cells

**FIGURE 4:**
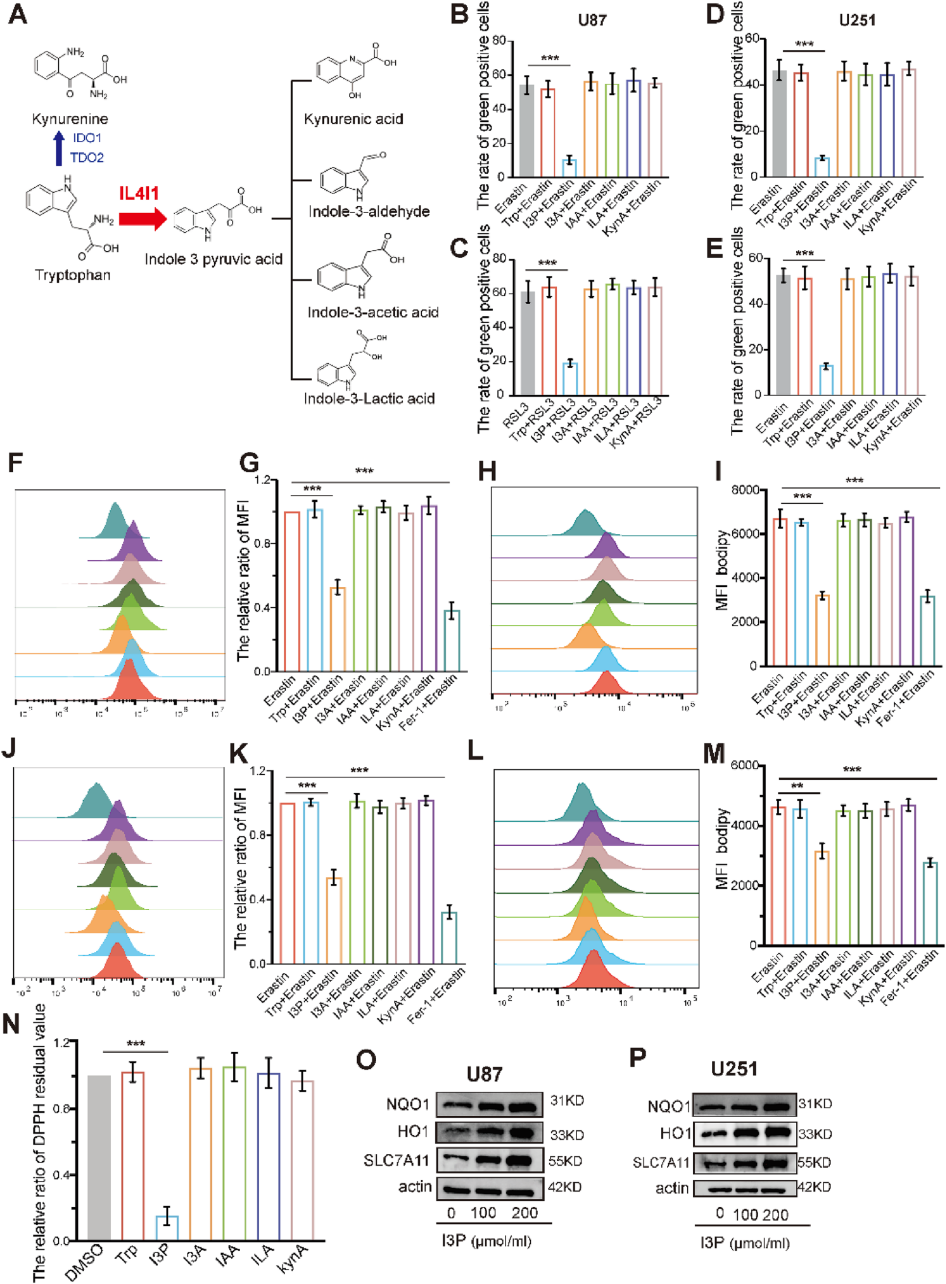
IL4I1‐driven tryptophan metabolites suppress ferroptosis. (A) Simplified diagram of the IL4I1‐mediated TRP catabolism pathway. (B–E) Quantification of cell death in U87 and U251 cells treated for 24 h with 10 mM of Erastin (B, D) or 1 mM of RSL3 (C, E) in the presence of 200 mM of TRP, I3P, KYNA, I3A, ILA, and IAA. (F–M) I3P block ROS and lipid peroxidation accumulation induced by 24 h of Erastin treatment in U87 cells (F–I) and U251 cells (J–M). ROS and lipid peroxidation quantification were determined by flow cytometry using the H2DCFDA and C11‐BODIPY probe, respectively. Fer‐1 was added as a positive control. (N) Cell‐free scavenging activity of 200 mM of each metabolite determined by changes in the absorbance at 517 nm of the stable radical DPPH relative to water control. (O, P) Effects of I3P on the protein levels of ferroptosis‐related genes in GBM cells. All error bars represent standard deviation. *p < 0.05, **p < 0.01, ***p < 0.001.

As shown in a previous study, IL4I1 played a more crucial role in the malignant process, compared with IDO or TDO, by mediating tryptophan catabolism [20]. Thus, we next explored which of the IL4I1 driven tryptophan metabolites had a more important role in ferroptosis resistance. Since classical theory holds that IL4I1 regulates the AHR pathway via I3P derivatives rather than via I3P itself, we added I3P and its derivatives, including I3A, IAA, ILA, and KynA (Figure 4A) into ferroptosis inducer‐treated U87 and U251 cells and examined cell viability after 24 h. Interestingly, we observed that addition of I3P maintained cell viability after 24 h of exposure to the ferroptosis inducers, indicating that I3P rather than its derivatives potently weakened ferroptosis (Figure 4B–E, Figure S5A–D). Based on the result that I3P significantly protected against ferroptosis, we next examined the level of ROS and lipid peroxidation in erastin‐ and RSL3‐treated cells. Consistent with previous results, only I3P decreased the level of ROS in U87 and U251 cells after treatment with ferroptosis inducers (Figure 4F,G,J,K; Figure S6A,B; and Figure S7A,B). C11‐BODIPY results also revealed that I3P rather than other I3P‐derived metabolites inhibited lipid peroxidation (Figure 4H,I,L,M; Figure S6C,D; and Figure S7C,D). Since a previous study provided evidence that I3P was a free radical scavenger [33], we detected the radical scavenging activity of each metabolite at 100 mM using the radical DPPH (Figure 4N) and found that only I3P scavenged the free radical. Moreover, I3P protected more efficiently against erastin‐induced ferroptosis rather than against RSL3‐induced ferroptosis. Thus, I3P might have a dual action in inhibiting ferroptosis, including the regulation of ferroptosis‐related genes and direct free radical scavenging. Consistent with a previous study [34], we found that I3P upregulated SLC7A11, NQO‐1, and HMOX‐1 (Figure 4O,P), while I3P‐derived metabolites could not influence the expression of anti‐ferroptosis‐related genes (Figure S8A–H). Overall, among the IL4I1‐mediated Trp metabolites, only I3P inhibited ferroptosis by regulating related genes and directly scavenging free radicals in GBM cells.

### 3.5 I3P‐Induced Redox‐Protective Gene Expression by Regulating Nrf2 Expression

**FIGURE 5:**
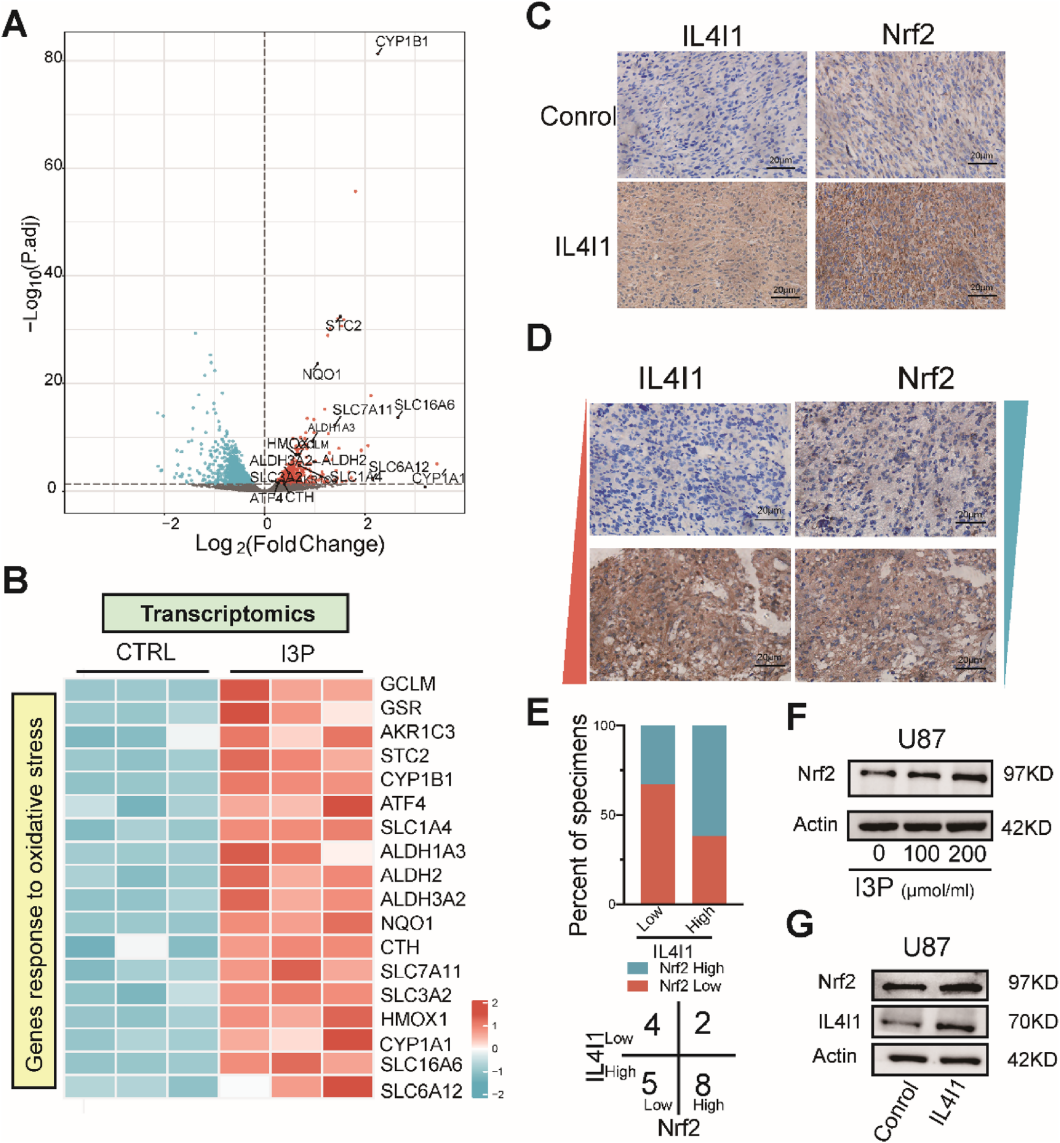
I3P‐induced redox‐protective gene expression by regulating Nrf2 expression and translocation. (A) Schematic depicting the volcano plots showing overall changes in the transcriptome in untreated versus I3P‐treated U87 cells. (B) Heatmap of selected genes that were significantly differentially expressed after 24 h of I3P treatment (adjusted *p* value < 0.05). (C) IHC analysis of IL4I1 and Nrf2 in mouse tumour sections. Scale bars, 20 μm. (D, E) Representative images of IHC staining showed IL4I1 expression positively correlated with Nrf2 in the same location. Scale bars, 20 μm. All error bars represent standard deviation. (F, G) Effects of I3P and IL4I1 overexpression on the protein levels of Nrf2 in U87 cells. *p < 0.05, **p < 0.01, ***p < 0.001.

To investigate how I3P reactions were involved in protecting cell against ferroptosis, RNA‐seq analysis was performed on U87 cells treated with I3P for 24 h. Among all DEGs, the expression of typical AHR pathway‐related genes including CYP1B1 and CYP1A1 was upregulated whereas other genes were regulated by the typical AhR pathways. Moreover, the expression of a series of mRNAs involved in cellular stress, especially in oxidative stress response, such as SLC7A11, NQO1, and HMOX‐1, (Figure 5A,B) was regulated. This result suggested that I3P might be involved in regulating Nrf2 pathway [35]. Consistently, the expression level of Nrf2 was increased when IL4I1 was overexpressed in vivo (Figure 5C). Furthermore, immunochemistry assays showed that IL4I1 was positively correlated with Nrf2 in human GBM samples (Figure 5D,E). Immunoblotting assays also showed that the protein levels of Nrf2 increased which mediated by I3P and overexpression of IL4I1 (Figure 5F,G; Figure S9C,D). However, other IL4I1‐mediated metabolites did not change the expression level of Nrf2 (Figure S8A–H).

### 3.6 I3P Decreased the Ubiquitination and Increased Nuclear Translocation of Nrf2 by Binding With Nrf2

**FIGURE 6:**
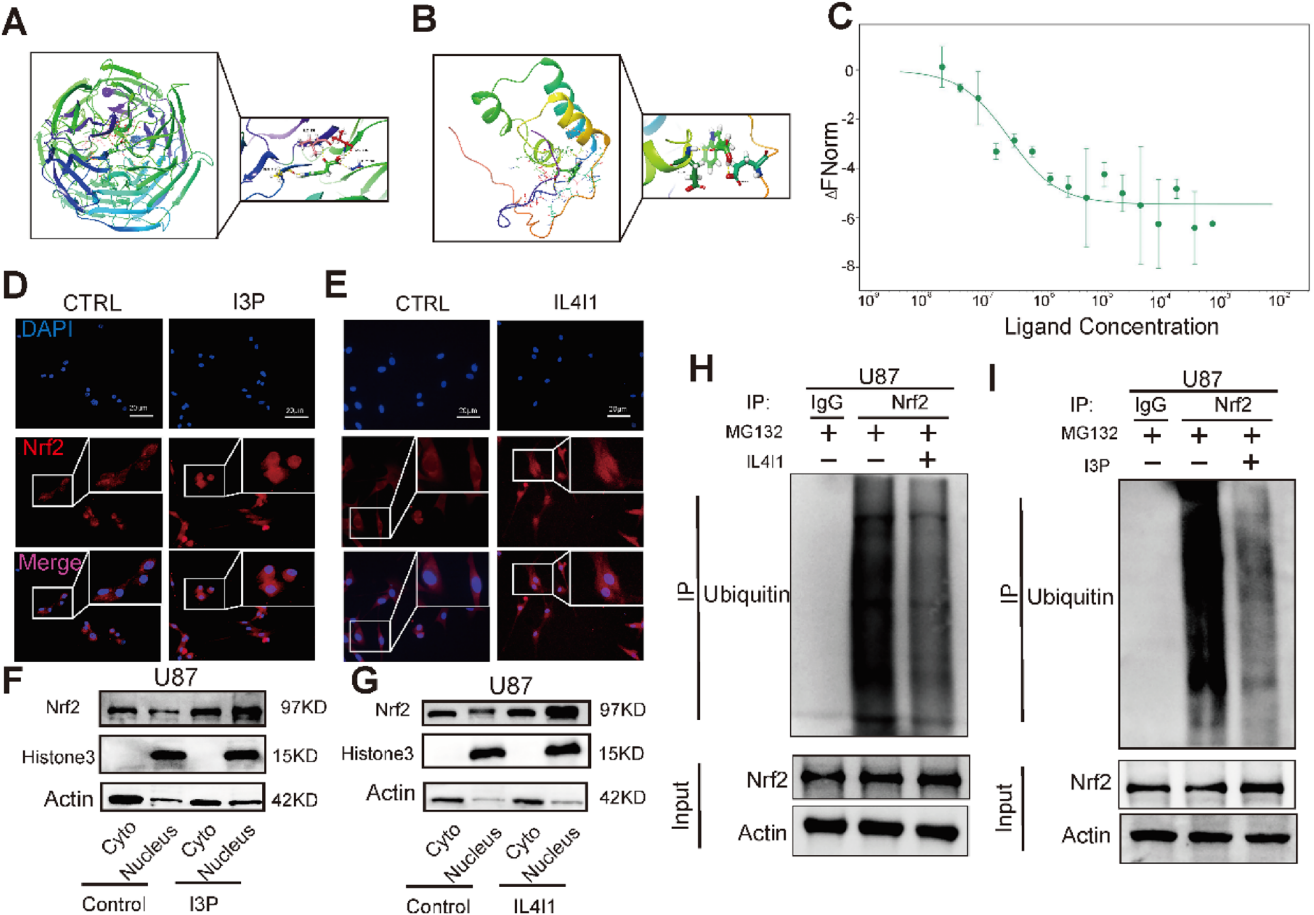
I3P decreased the ubiquitination and increased nuclear translocation of Nrf2 by binding with Nrf2. (A) Computational docking model between I3P and Neh2. (B) Computational docking model between I3P and Neh1. (C) MST of I3P and full length Nrf2. (D, E) The effects of I3P treatment and IL4I1 overexpression on the intracellular localisation of and expression of Nrf2 were assessed by immunofluorescence in U87 cells. (F, G) Effect of I3P treatment and IL4I1 expression on Nrf2 nuclear‐cytoplasmic translocation. (H, I) Effect of I3P treatment and IL4I1 expression on Nrf2 ubiquitination.

Based on the above results, we found that although there was no significant increase in Nrf2 at the transcriptional level, there was a significant increase at the protein level. Further, we suspected that I3P might interact with Nrf2 to activate its function. Multifaceted regulatory mechanisms for Nrf2 protein levels or activity have been mapped to the functional domains of Nrf2, Neh1‐7 [36]. We found that the I3P may bind to Neh2 (PDB: 3ZGC) which was a domain related ubiquitination of Nrf2 by a docking model with a score (−7.915) (Figure 6A) and I3P may also bind to Neh1 (PDB: 2LZ1) which was a domain related transcriptional activity of Nrf2 by a docking model with a score (−5.616) (Figure 6B). To further investigate whether I3P directly interacts with Nrf2, thus, we performed MST to study the relationship between and human recombinant protein NRF2. It was observed that there was a significant ligand‐induced fluorescence change, which demonstrated that I3P can directly interact with Nrf2 (Figure 6C). Subsequently, immunofluorescence assay results showed that the nuclear translocation and expression of Nrf2 were enhanced in both I3P‐treated and IL4I1‐overexpressing cells (Figure 6D,E; Figure S9G,H; and Figure S9A,B). Consistently, we performed nuclear‐cytoplasmic extraction of proteins and found that IL4I1 overexpression increased the nuclear and cytoplasmic Nrf2 levels in U87 and U251 cells (Figure 6F,G; Figure S9I,J; and Figure S9E,F). We also performed co‐immunoprecipitation assays and found that the ubiquitination level of Nrf2 was significant lower in both I3P‐treated and IL4I1‐overexpressing cells (Figure 6H,I). Thus, this explained why the protein level of Nrf2 was significantly upregulated while its mRNA level was only slightly upregulated. Overall, our findings indicated that tryptophan‐driven I3P enhanced the nuclear translocation and decreased the ubiquitination of Nrf2 by directly binding with Nrf2.

### 3.7 IL4I1 Protected Against Ferroptosis Independent of AHR but Dependent on Tryptophan and Nrf2 Activation

**FIGURE 7:**
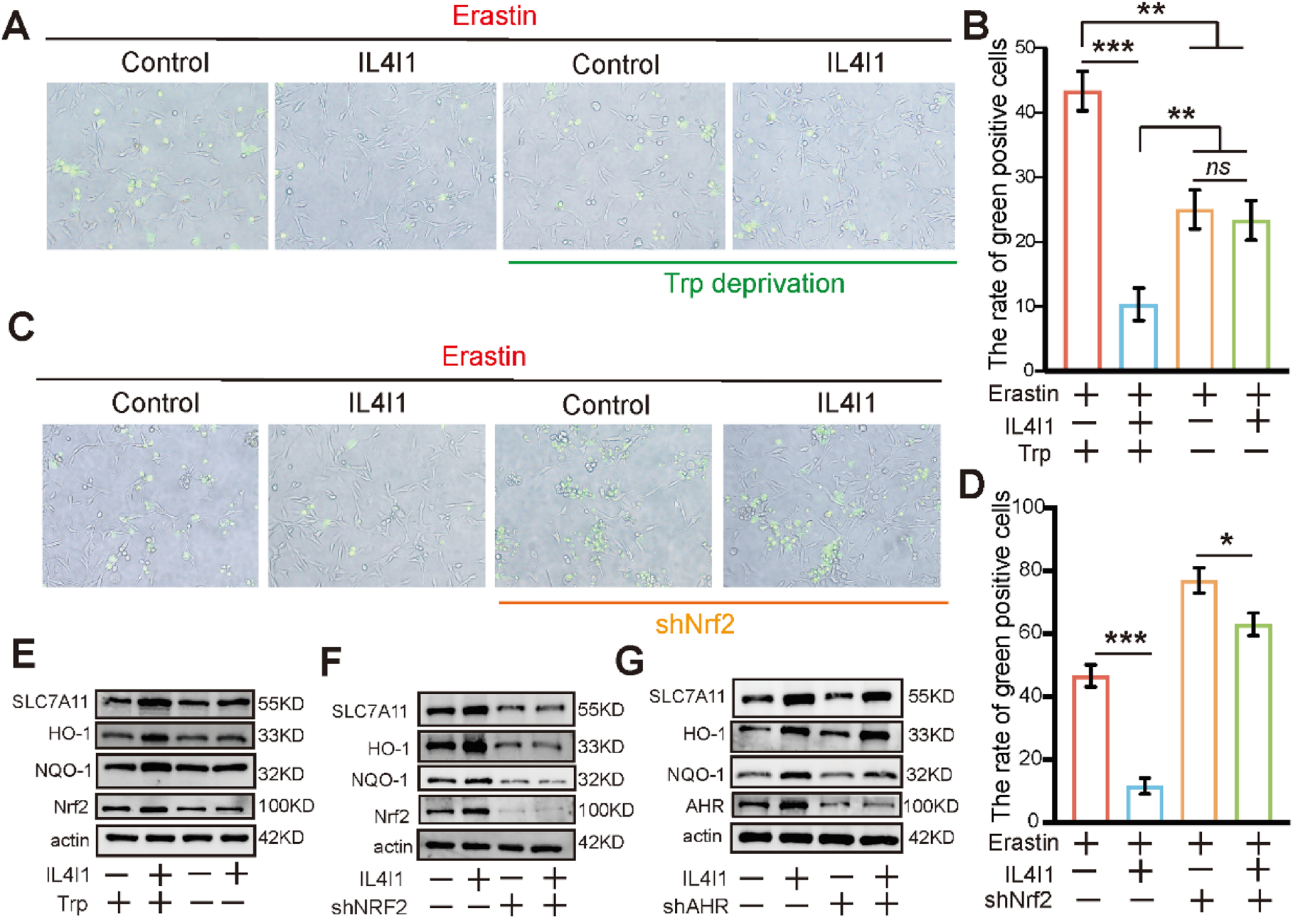
IL4I1 protected against ferroptosis independent of the AHR but dependent on tryptophan and activation of Nrf2 in vitro. (A, B) Presence and quantification of cell death in control and Il4I1 overexpression U87 cells with erastin in normal and TRP‐deprived media. (C, D) Presence and quantification of cell death in IL4I1 overexpression U87 cells and Nrf2 knockdown with erastin. (E) Effects of IL4I1 overexpression and TRP‐deprivation on the protein levels of Nrf2, NQO‐1, HO‐1, and SLC7A11 in U87 cell. (F) Effects of IL4I1 overexpression and Nrf2 knockdown on the protein levels of Nrf2, NQO‐1, HO‐1, and SLC7A11 in U87 cell. (G) Effects of IL4I1 overexpression and AHR knockdown on the protein levels of Nrf2, NQO‐1, HO‐1, and SLC7A11 in U87 cell. All error bars represent standard deviation. *p < 0.05, **p < 0.01, ***p < 0.001.

We further investigated whether the function of IL4I1 was dependent on tryptophan catabolism and Nrf2 activation. First, to explore the correlation between IL4I1 and tryptophan, we controlled the amount of tryptophan in the cell media. Interestingly, after adding erastin in the cell media, the overexpressed IL4I1 could not protect against ferroptosis in tryptophan‐free medium, indicating that IL4I1 function was tryptophan‐dependent. However, cell death suppression was also observed in tryptophan‐free media compared with normal media (Figure 7A,B). Consistently, the protein level of Nrf2 and its target genes did not change (Figures 7E and S10A). The explanation for this phenomenon is that tryptophan restriction might inhibit the proliferation and translation of cells which could decrease the consumption of cystine, which could generate GSH to suppress ferroptosis [17, 32, 37]. Next, we sought to determine if IL4I1‐mediated anti‐ferroptosis was Nrf2‐dependent. We knocked down Nrf2 in the experimental group and treated the experimental and control groups with erastin. Cell death remarkably increased in the Nrf2 knock‐down group, IL4I1‐overexpression was still able to prevent ferroptosis in tumour cells, however, this ability was obviously weakened when compared with that of the Nrf2 expressing group (Figure 7C,D). Western blotting showed that the expression of target genes was also downregulated in the Nrf2 knockdown group (Figure 7F, Figure S10B). It is well known that the AHR signalling could be activated by I3P and kynurenine derived from tryptophan [38]. Thus, we next determined if the expression of anti‐ferroptosis genes could be influenced by the AHR pathway. Western blot results revealed that the anti‐ferroptosis genes were still upregulated in the IL4I1‐overexpression group after AHR inhibition (Figure 7G, Figure S10C), indicating that IL4I1 suppressed ferroptosis independent of the AHR pathway.

**FIGURE 8:**
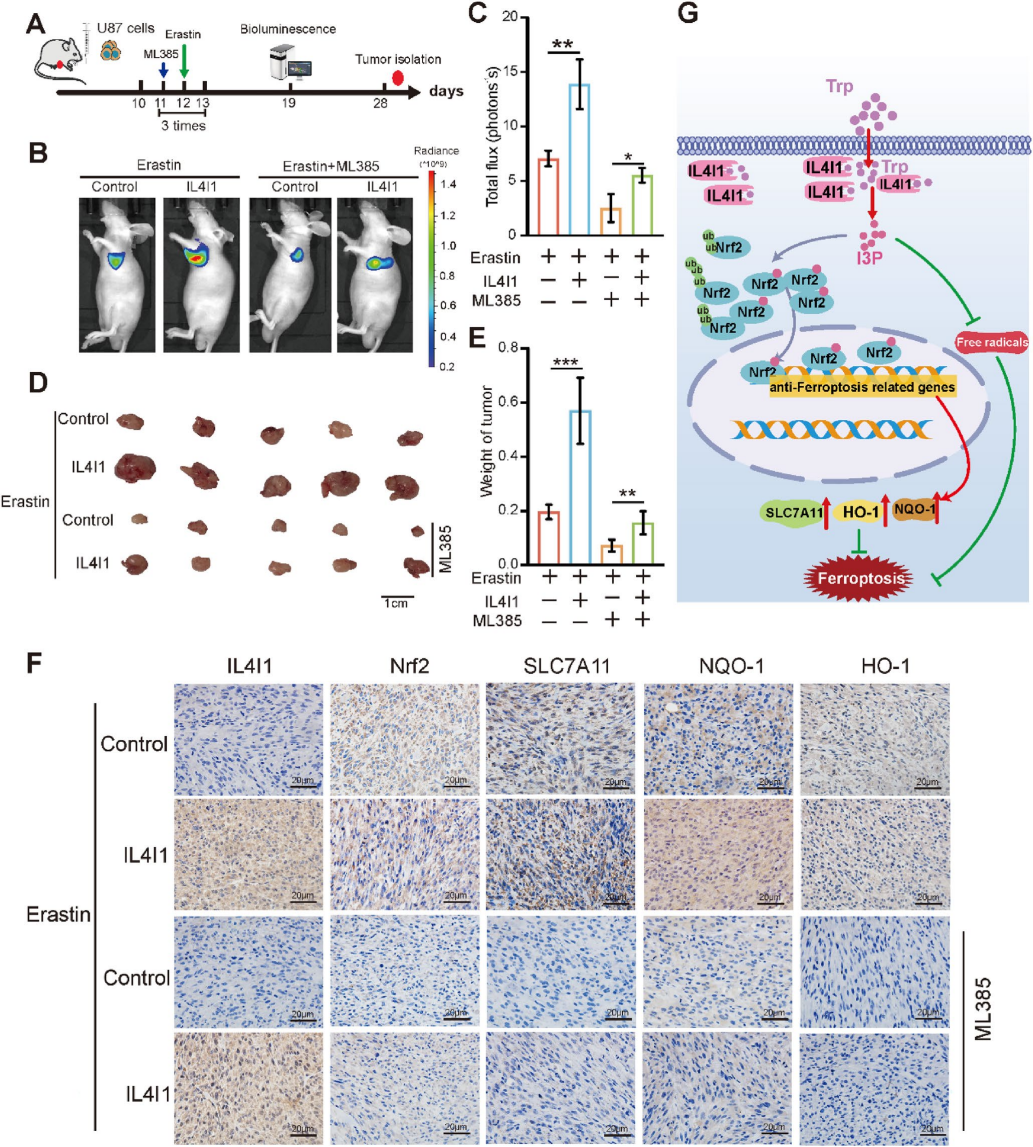
IL4I1 protected against ferroptosis dependent on activation of Nrf2 in vivo. (A) The schematic diagram showed treatment with mouse xenograft model. (B) Luciferase fluorescence of mouse xenografts treated with erastin and ML385 were detected by an IVIS at 19th day after tumour injection. (C) Quantification of luciferase fluorescence (*n* = 3). (D, E) Representative images and quantification of mouse tumours. (F) IHC analyses of IL4I1, Nrf2, SLC7A11, NQO‐1, and HOMX‐1 in mouse tumour sections. Scale bars, 20 μm. (G) Mechanistic model for IL4I1 regulation of cell ferroptosis in GBM. All error bars represent standard deviation. *p < 0.05, **p < 0.01, ***p < 0.001.

We then verified these results in vivo. Considering the blood–brain barrier blockade of drug delivery and physiologic instability of erastin and ML385—a specific inhibitor of Nrf2 in vivo, we constructed a subcutaneously implanted tumour model in nude mice. The operating steps are shown in Figure 8A. Bioluminescence imaging showed that after three treatments with erastin and ML385, the tumour volume of the IL4I1 overexpression group was larger compared with that of the control group, and that the tumour volume of the IL4I1 overexpression group treated with both erastin and ML385 was smaller compared with that of the IL4I1 overexpression group only treated with erastin (Figure 8B,C). The same result was shown by measuring the weight of tumours isolated from the model mice (Figure 8D,E). Furthermore, IHC assays showed that Nrf2 and its target genes were significantly upregulated in the IL4I1 overexpression group compared with the other groups (Figure 8F), indicating that IL4I1 regulated ferroptosis‐related genes in an Nrf2‐dependent way. However, despite that IL4I1‐overexpression had not resulted in upregulation of ferroptosis‐related genes in the ML385‐treated group, the tumour volume was significantly increased (Figure 8C–F). This strongly supported our previous findings that indicated that IL4I1 acts against ferroptosis via some Nrf2‐independent pathways. Overall, we verified that IL4I1 protected against ferroptosis independent of AHR but dependent on tryptophan and Nrf2 activation in vitro and in vivo.

In the current study, we revealed that IL4I1 elevated the expression of Nrf2 and promoted its nuclear translocation to promote the transcription of anti‐ferroptosis‐related genes, HO‐1, SLC7A11, and NQO‐1, through I3P rather than other tryptophan metabolites. Furthermore, I3P scavenged free radicals directly. Thus, we identified IL4I1 as novel anti‐ferroptosis regulator in GBM (Figure 8G).

When ferroptosis was first time put forward and identified as an iron‐dependent form of nonapoptotic cell death [39], amino acid metabolism, which was always thought to participate in protein synthesis and energy supply to support cancer cells [40], was revealed to play an important role in the regulation of ferroptosis [41]. Recently, increasing evidence confirmed its important function in ferroptosis. For instance, cystine acted as an antioxidant by itself and played an important role in transforming GSH/GSSG to inhibit ferroptosis [42]. Compared with the direct regulation of ferroptosis by cystine, branched‐chain amino acids mediated by BCAT regulated ferroptosis indirectly by changing the level of metabolites [43]. Similarly, we found that IL4I1 catabolism of tryptophan into I3P scavenged free radicals directly to suppress ferroptosis, however, other tryptophan‐derived metabolites including I3A, IAA, ILA, and kynA did not have this function. Moreover, we demonstrated that the expression of anti‐ferroptosis‐related genes was changed. It is established that complicated regulatory networks regulate ferroptosis and that the change of any key node might trigger or inhibit ferroptosis [34]. System xc^−^cystine/glutamate antiporter transport system composed of SLC7A11 and SLC3A2 was found to be responsible for exchanging intracellular glutamate via cystine uptake to provide GSH [44]. Further, GPX4 reduced GSH to convert lipid hydroperoxides to lipid alcohols, which decreased lipid peroxidation [45]. Thus, the xCT/GPX4 axis plays a significant role in protecting against ferroptosis. In our study, IL4I1 increased the SLC7A11 expression, thus enhancing the biosynthesis of GSH to protect against erastin‐induced ferroptosis. Interestingly, overexpression of IL4I1 also weakened RSL3‐induced ferroptosis although the expression of GPX4 did not change. ROS scavenging and the regulation of other genes might be potential underlying mechanisms.

Our RNA‐seq data from I3P‐treated cells showed that NQO‐1, HO‐1, and SLC7A11 were upregulated. Furthermore, we detected the mRNA and protein expression levels of these two genes in IL4I1 overexpression cells and the results were verified using the RNA‐seq data. SLC7A11, NQO‐1, and HO‐1 were important genes in the regulation of anti‐oxidative stress and Nrf2 target genes [46, 47]. Nrf2 was defined as a key transcription factor protecting cells against oxidative stress [48] and there were different domains including Nrf2‐ECH homology (Neh)1–7 were responsible for multiple functions. For instance, Neh1 was the cap'n'collar‐leucine zipper domain, promoted target gene transcription by recognising and binding with anti‐oxidant response elements [49]. Interestingly, we found that I3P might bind with the Neh2 domain of Nrf2. As is well known, KEAP1 binds NRF2 as a dimer, interacting through its C‐terminal Kelch domain with the DLG and ETGE motifs located in the Neh2 domain of Nrf2, promoting the degradation of Nrf2 via ubiquitylation [50]. Ubiquitylation regulated gene expression on the protein level instead of the transcriptional level, probably explaining the increase in the protein level of Nrf2 rather than the mRNA level. Thus, we revealed a new way that IL4I1 could promote the activation of Nrf2, that is, via I3P binding with the Neh2 domain. This was different from the mechanism presented in a previous study, in which IL4I1 activated the AHR signalling pathway through I3P and I3A binding with AHR [20].

Tryptophan‐derived metabolites activate AHR, promote tumour progression, and regulate the TME in different tumours, including GBM [18]. Du et al. found that the expression levels of IDO1 and TDO2 were higher in gliomas compared with those in normal brain tissue and positively correlated with the grade of gliomas. Furthermore, these TCEs enhanced the invasion and migration of glioma cells by promoting the expression of AQP4 via the kynurenine/AHR signalling pathway [51]. Moreover, tryptophan catabolism provided a precursor for the generation of NAD, which played a crucial role in DNA repair, thereby enhancing glioma cell resistance to chemoradiotherapy [52]. In the GBM TME, tryptophan‐generated kynurenine promoted the formation of an immunosuppressive TME by regulating the phenotype of TAMs and inhibiting the proliferation of CD8 + T cells [53]. Thus, inhibiting the TCEs represents a new promising strategy for GBM treatment. Compared with classical TCEs including IDO1 and TDO2, IL4I1 plays a more crucial role in regulating of tryptophan catabolism [20]. We first verified the IL4I1 mRNA and protein levels in patients with different glioma grades. Our results suggest that IL4I1 acts as an important biomarker to stratify patients for diagnosis and therapy. Further, the IL4I1 protein level in patient serum and the glioma TME will also be detected. In previous studies, the function of tryptophan metabolites focused on regulating immune cells, such as promoting the activation or differentiation of regulatory T cells [54] and regulation of TAMs [55]. Recently, it has been revealed that kynurenine and derived metabolites are not only potent neuro‐ and immunomodulators but are also dependent on the redox state and may serve as ROS scavengers or generators [17, 56, 57]. This is the first study to reveal that I3P, generated by IL4I1‐mediated tryptophan catabolism, regulates anti‐oxidative stress via Nrf2 and serves as a ROS scavenger to suppress ferroptosis in GBM. Moreover, we verified that the downstream metabolites of I3P did not have this function.

## 4 Conclusion

In summary, our study has highlighted a novel anti‐ferroptosis regulator‐IL4I1 by regulating Nrf2 and serving as a ROS scavenger in GBM. Besides, we found that the ubiquitination of Nrf2 could be attenuate by I3P binding with Nrf2 directly. Furthermore, we also identified IL4I1 as a potential prognostic biomarker and provided a novel anti‐tumour therapeutic target for GBM.

## Author Contributions

Qianxue Chen, Qingsong Ye, and Yangzhi Qi contributed the designed the project and revised the manuscript. Yang Xu, Yu Hong, and Tengfeng Yan performed the experiments and wrote the manuscript. Qian Sun and Fanen Yuan analysed data. Shanwen Liang and Liguo Ye were responsible to RNA‐seq data and bioinformation analysis. Rongxin Geng supported clinical information and analysis. All authors have read and approved the final version of this manuscript.

## Conflicts of Interest

The authors declare no conflicts of interest.

## Supporting information


**DATA S1** Supplementary figures.

**DATA S2** Supplementary tables.

## Data Availability

The data that support the findings of this study are available on request from the corresponding author. The data are not publicly available due to privacy or ethical restrictions.
