# Supplementary material for: Tryptophan Metabolic Enzyme IL4I1 Inhibits Ferroptosis by Decreasing Ubiquitination of Nrf2 via I3P in Glioblastoma
Source: Cell Prolif. 2025 Mar 12;58(6):e13816. doi: 10.1111/cpr.13816 (PMC12179557; doi:10.1111/cpr.13816)
Supplement: Supplementary file 1 — DATA S1 Supplementary figures. [file CPR-58-e13816-s001.docx]

**Supplementary Materials**

**Tryptophan metabolic enzyme IL4I1 inhibits ferroptosis by decreasing ubiquitination of Nrf2 via I3P in glioblastoma**

Yang Xu^1,4#^, Yu Hong^1,4#^, Wei Xiong^2,5#^, Tengfeng Yan^1,3#^, Qian Sun^1,4^, Fanen Yuan^1,4^, Shanwen Liang^1,4^, Liguo Ye^1,4^, Rongxin Geng^1,4^, Yangzhi Qi^1,4*^, Qingsong Ye^2*^, Qianxue Chen^1*^


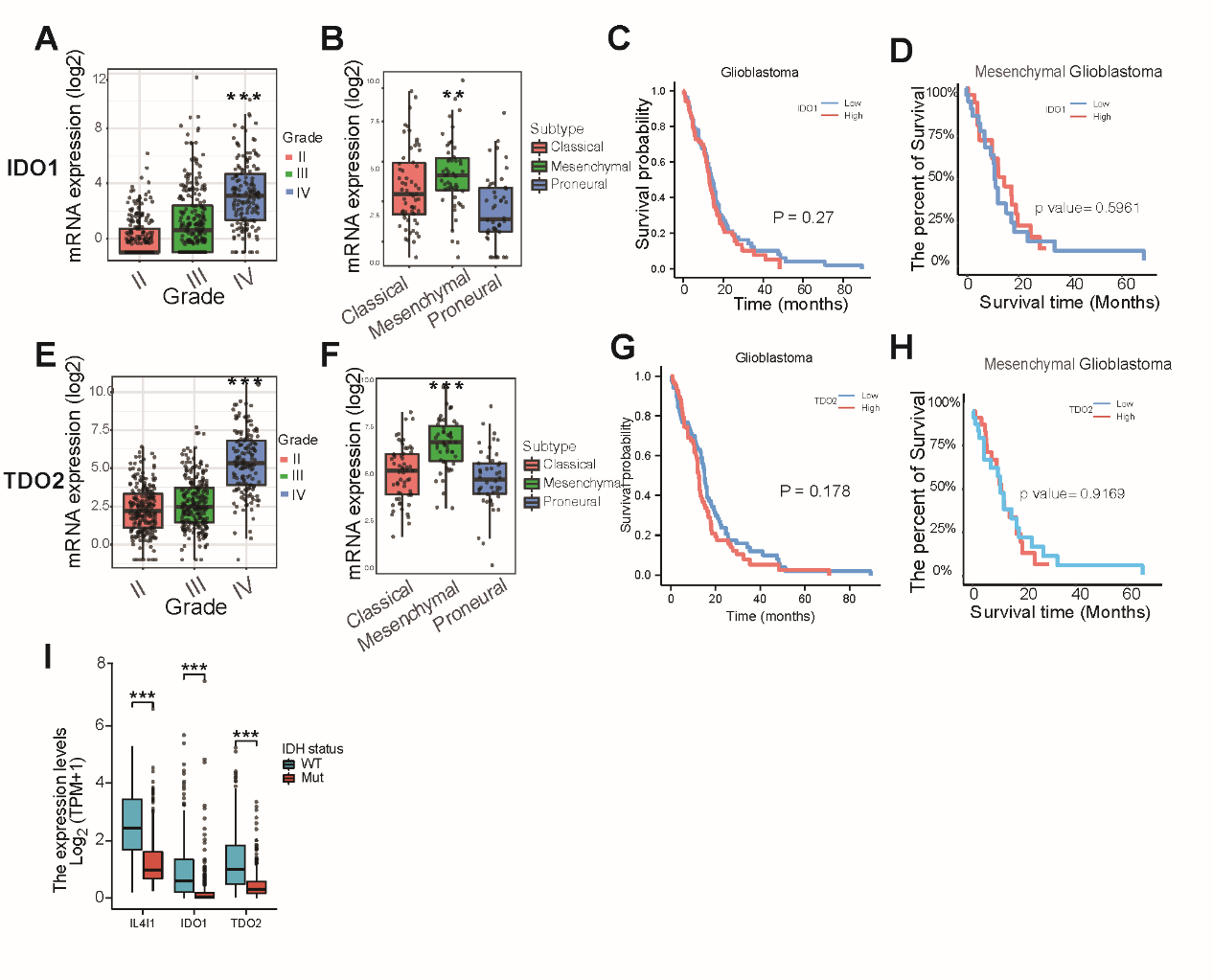


**FigureS1 IL4I1 was upregulated and related to poor outcomes in GBM according to TCGA database.** A, E. Expression of IDO1 and TDO2 in human gliomas according to the TCGA database. B, F. Comparison of IDO1 and TDO2 expression levels between GBM MES, PN, or CL subtypes. Boxplots indicate the median quartiles, with whiskers extending the minimum and maximum range. C, G Kaplan Meier curves of the overall survival of GBM patients in TCGA divided into groups of high and low IDO1 and TDO2 expression. D, H Kaplan Meier curves of the overall survival of MES type GBM patients in TCGA divided into groups of high and low IDO1 and TDO2 expression. I. The expression of IL4I1, IDO1 and TDO2 in IDH WT and mut type gliomas. The adjusted p value of the maximally selected log rank statistic for the cut-point search is shown. **P < 0.05, **P < 0.01, ***P <0.001.*


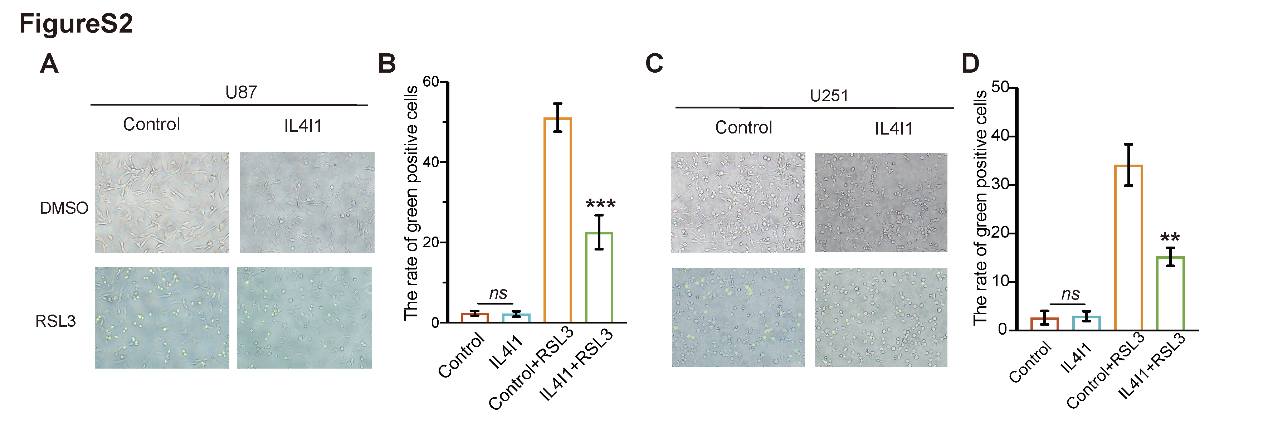


**FigureS2.** A-D. IL4I1 inhibited RSL3 induced ferroptosis in U87 cell and U251 cells according to Cell Tox green. All error bars represent standard deviation. **P < 0.05, **P < 0.01, ***P <0.001.*


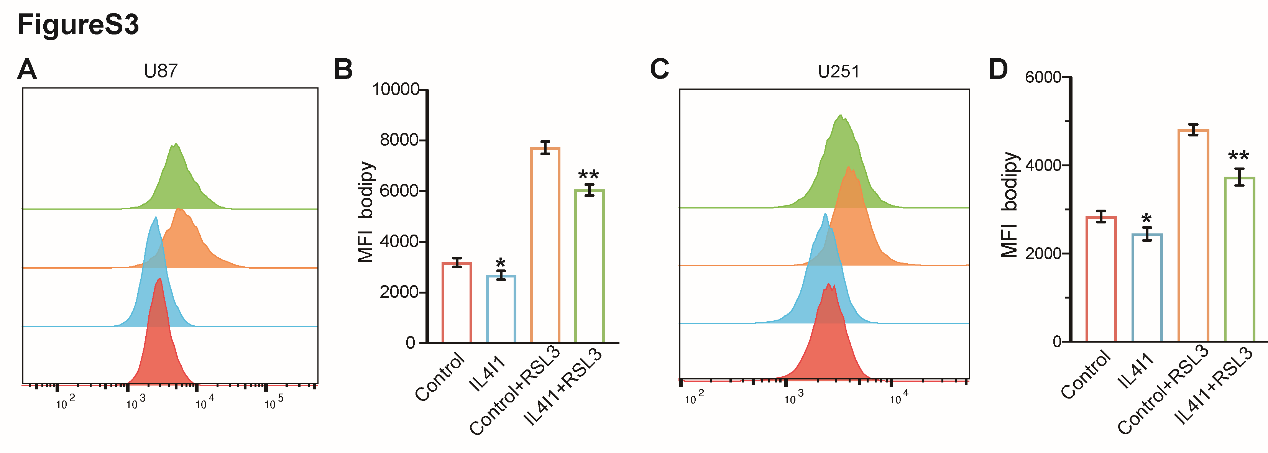


**FigureS3.** A-D. IL4I1 decreased lipid peroxidation induced by RSL3 determined by flow cytometry using C11-BODIPY in U87 and U251 cells. All error bars represent standard deviation. **P < 0.05, **P < 0.01, ***P <0.001.*


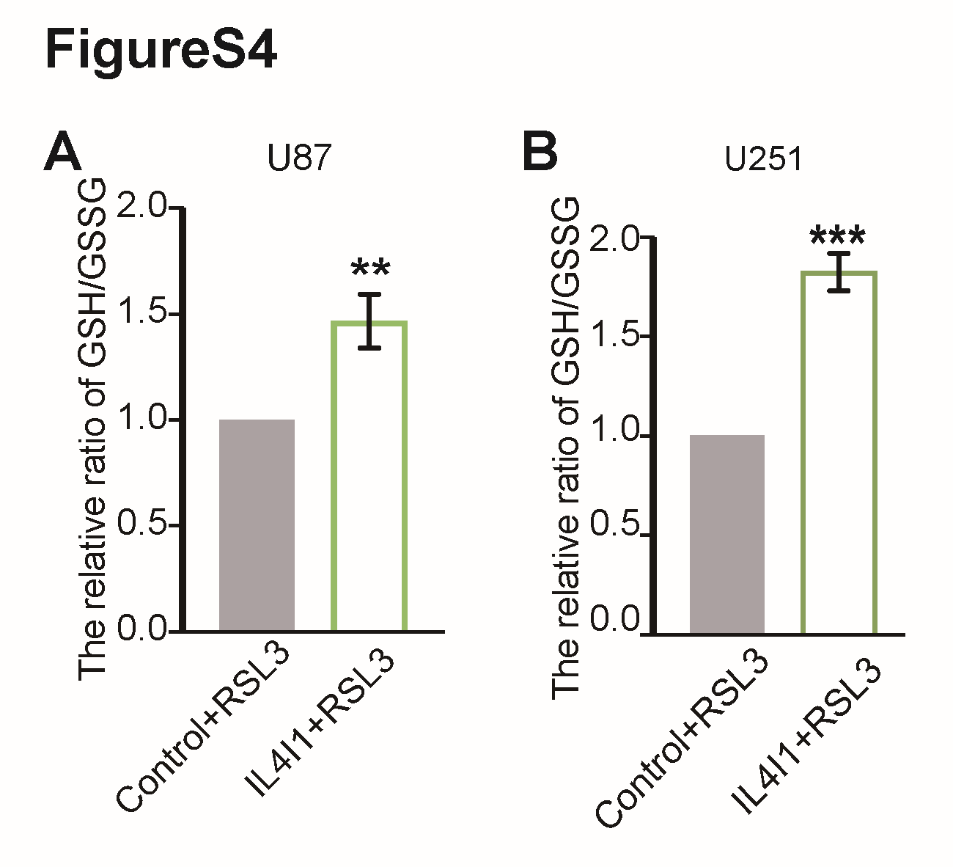


**FigureS4.** A, B IL4I1 increased GSH/GSSG ratio in U87 and U251 cells treated with Erastin for 24 hours relative to control. All error bars represent standard deviation. **P < 0.05, **P < 0.01, ***P <0.001.*


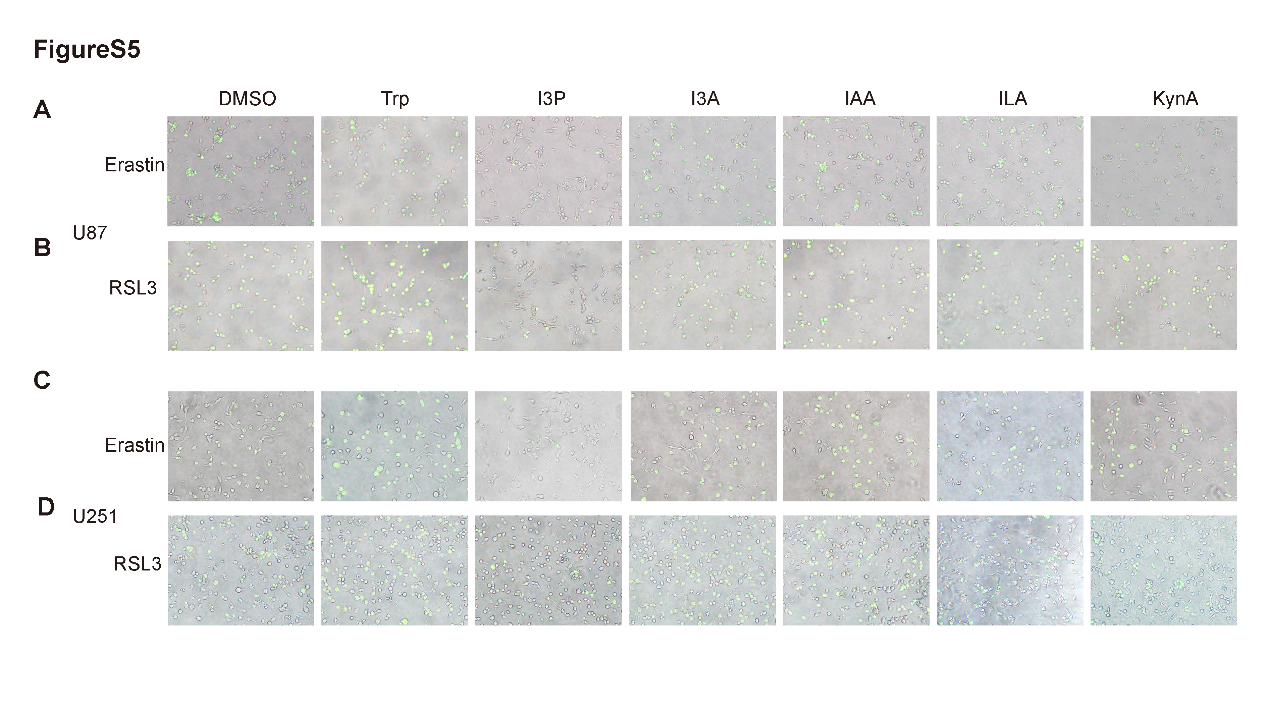


**FigureS5.** A-D. Quantification of cell death in U87 and U251 cells treated for 24 h with 10 mM of erastin (A, C) or 1 mM of RSL3 (B, D) in the presence of 200 mM of TRP, I3P, KYNA, I3A, ILA, IAA. E-L. E-L.


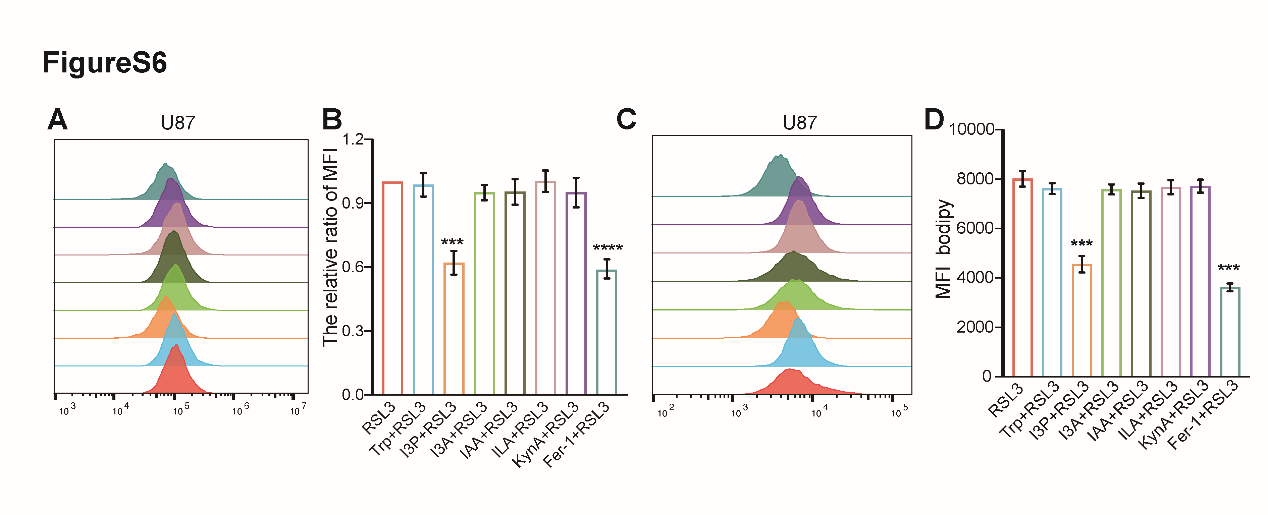


**FigureS6.** A-D. I3P block ROS and lipid peroxidation accumulation induced by 24 h of RSL3 treatment in U87 cells. ROS and lipid peroxidation quantification was determined by flow cytometry using the H2DCFDA and C11-BODIPY probe, respectively. Fer-1 was added as a positive control.


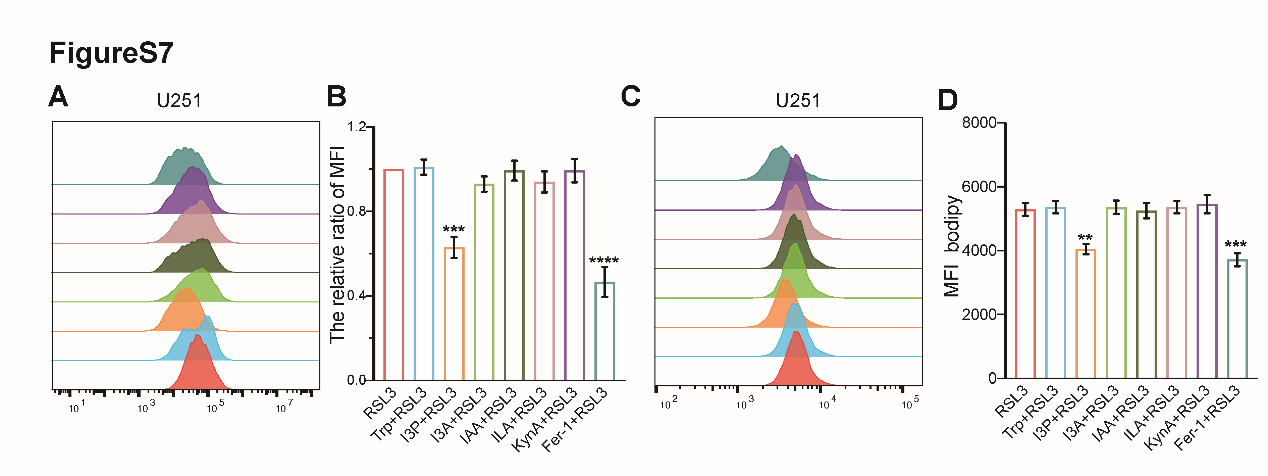


**FigureS7.** A-D. I3P block ROS and lipid peroxidation accumulation induced by 24 h of RSL3 treatment in U251 cells. ROS and lipid peroxidation quantification was determined by flow cytometry using the H2DCFDA and C11-BODIPY probe, respectively. Fer-1 was added as a positive control.


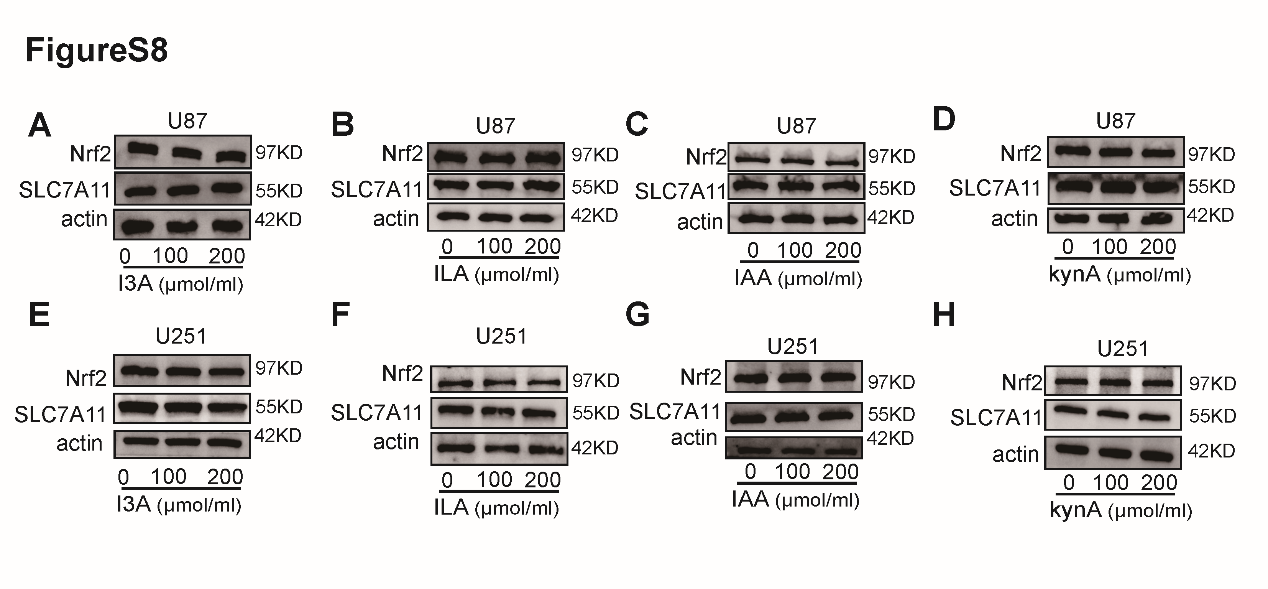


**FigureS8.** A-D. Effects of KYNA, I3A, ILA, IAA on the levels of Nrf2 and SLC7A11 in U87 cells. E-H. Effects of KYNA, I3A, ILA, IAA on the levels of Nrf2 and SLC7A11 in U251 cells.


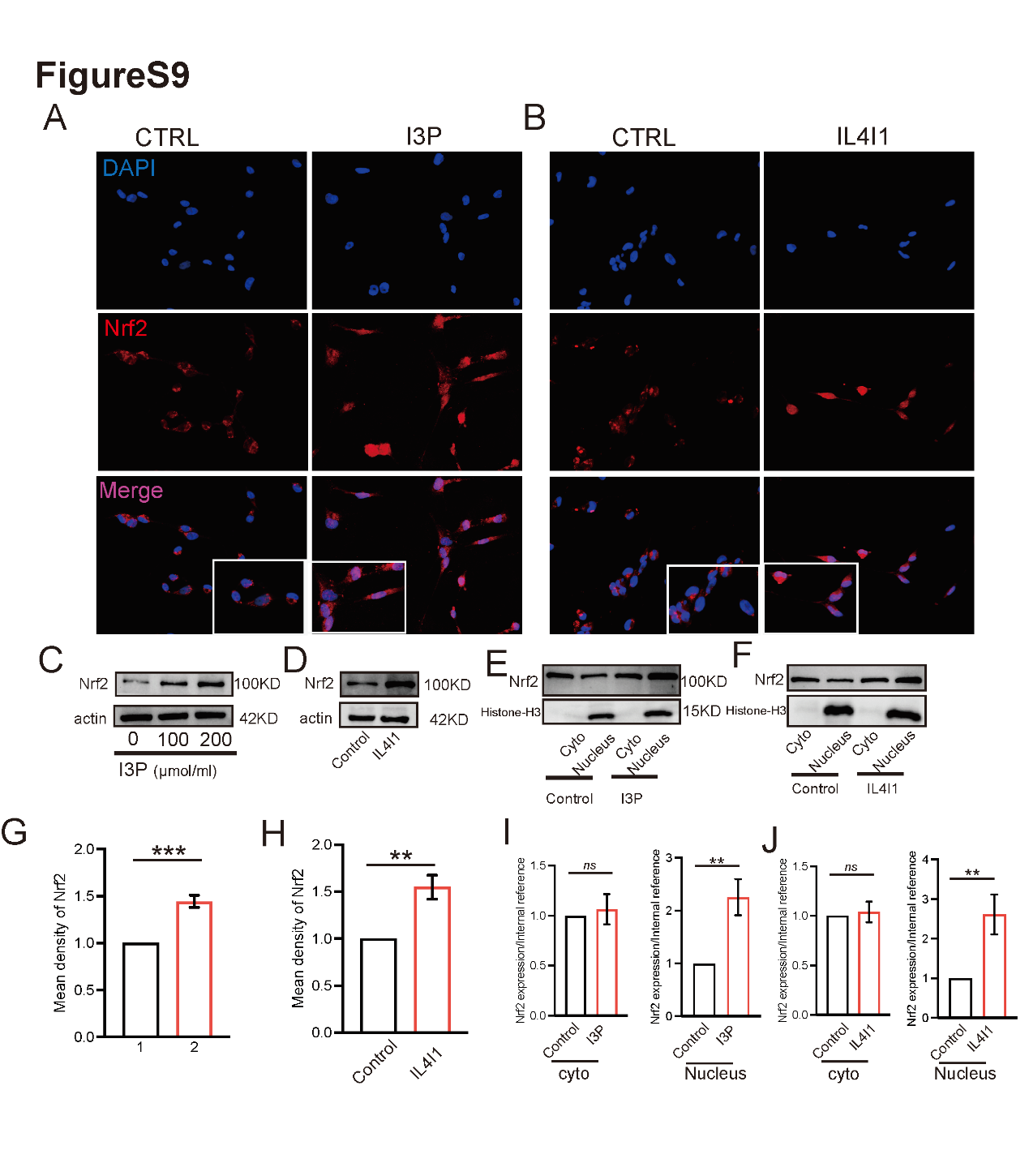


**FigureS9.** A, B. The effects of I3P treatment and IL4I1 overexpression on the intracellular localization of and expression of Nrf2 were assessed by immunofluorescence in U251 cells. C, D. Effects of I3P and IL4I1 overexpression on the protein levels of Nrf2 in U251 cells. E, F. Effect of I3P treatment and Il4I1 expression on Nrf2 nuclear-cytoplasmic translocation in U251 cells. G, H. Qualification of immunofluorescence of Nrf2 in I3P and IL4I1 overexpression U87 cells. I, J. Qualification of Nrf2 nuclear-cytoplasmic translocation in U87 cells treated with I3P and IL4I1 overexpression.

**
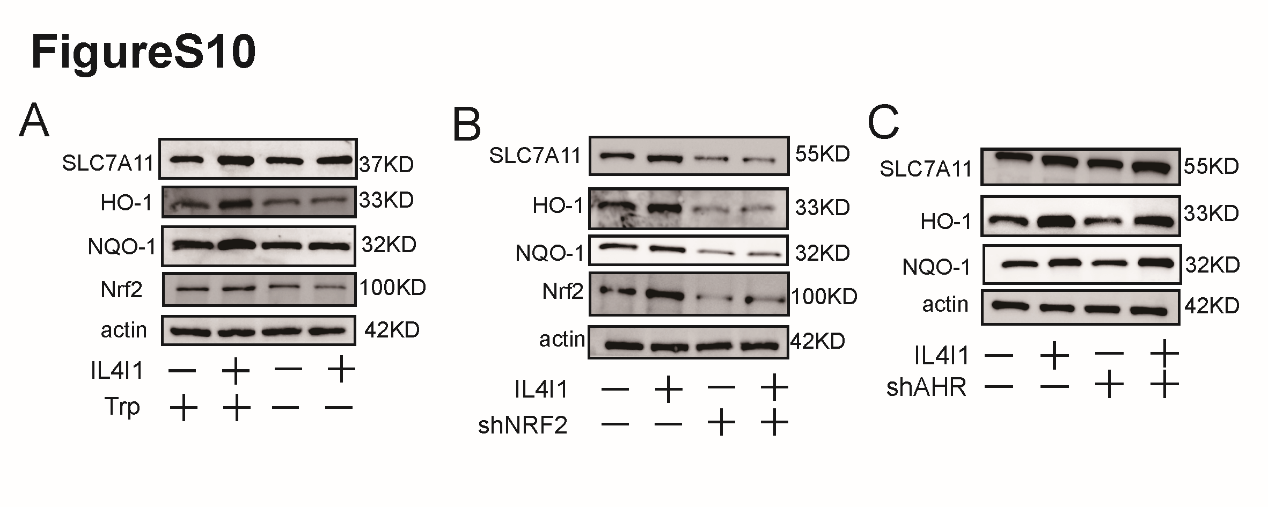
**

**FigureS10.** A. Effects of IL4I1 overexpression and TRP-deprivation on the protein levels of Nrf2, NQO-1, HO-1 and SLC7A11 in U251 cell. C. Effects of IL4I1 overexpression and Nrf2 knockdown on the protein levels of Nrf2, NQO-1, HO-1 and SLC7A11 in U251 cell. C. Effects of IL4I1 overexpression and AHR knockdown on the protein levels of Nrf2, NQO-1, HO-1 and SLC7A11 in U251 cell.
