# Supplementary figures and images for: Tryptophan Metabolic Enzyme IL4I1 Inhibits Ferroptosis by Decreasing Ubiquitination of Nrf2 via I3P in Glioblastoma
Source: Cell Prolif. 2025 Mar 12;58(6):e13816. doi: 10.1111/cpr.13816 (PMC12179557; doi:10.1111/cpr.13816)

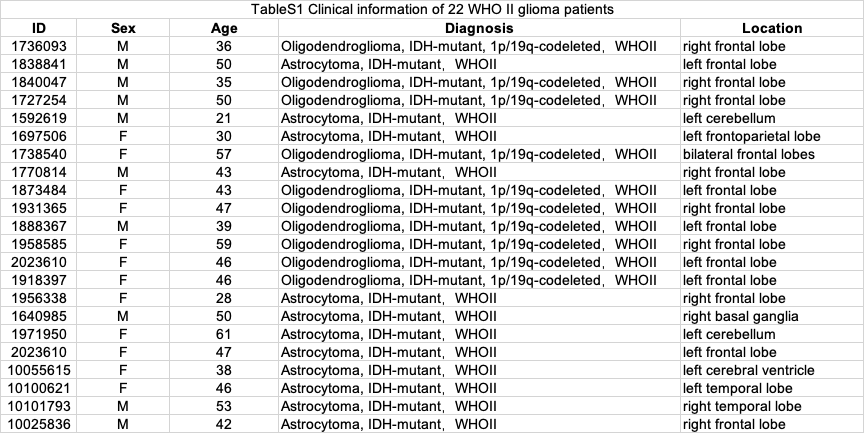


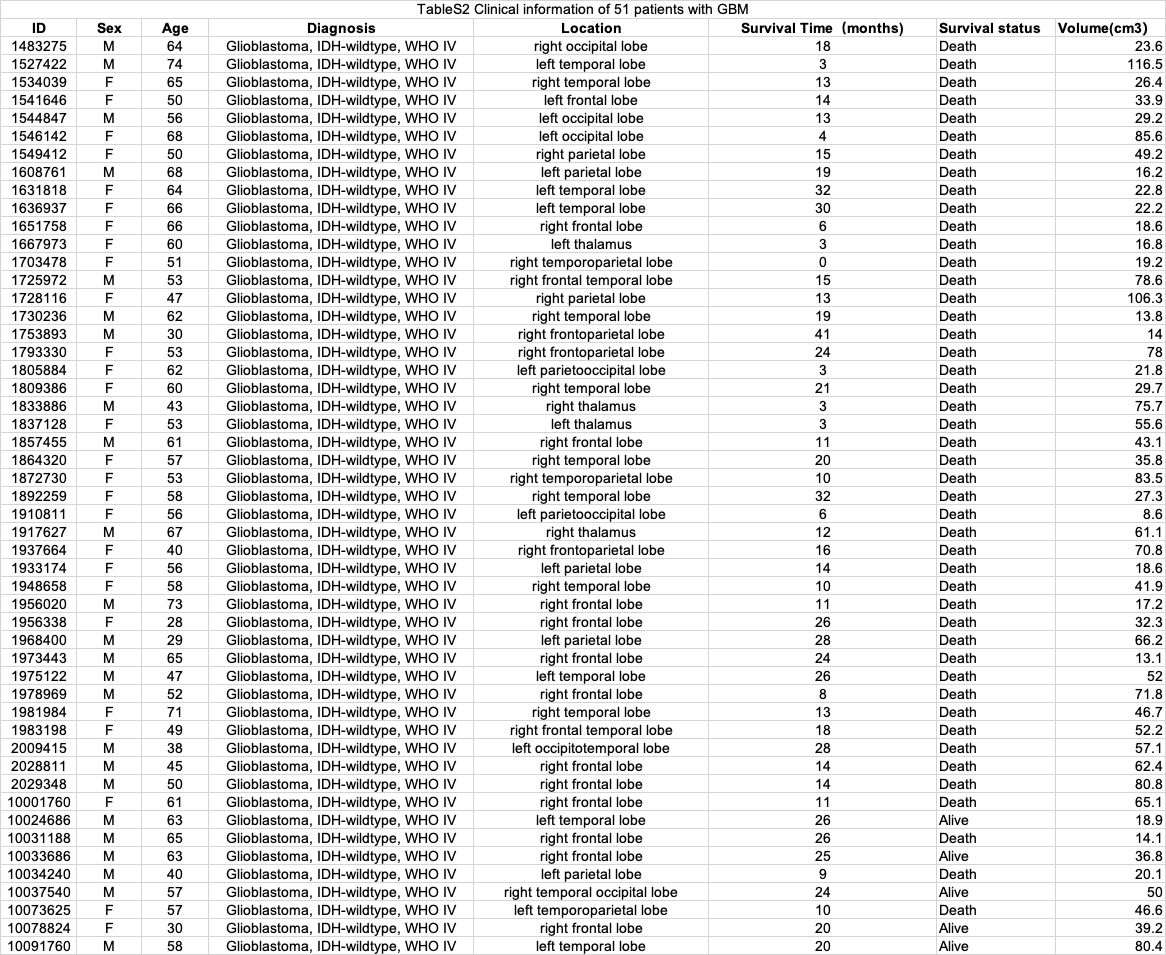


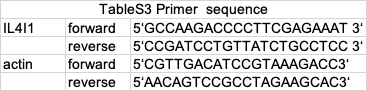

Supplement: Supplementary file 2 — DATA S2 Supplementary tables. [file CPR-58-e13816-s002.docx]
